# Lipid droplet‐mediated scavenging as novel intrinsic and adaptive resistance factor against the multikinase inhibitor ponatinib

**DOI:** 10.1002/ijc.32924

**Published:** 2020-03-02

**Authors:** Bernhard Englinger, Anna Laemmerer, Patrick Moser, Sebastian Kallus, Clemens Röhrl, Christine Pirker, Dina Baier, Thomas Mohr, Laura Niederstaetter, Samuel M. Meier‐Menches, Christopher Gerner, Lisa Gabler, Johannes Gojo, Gerald Timelthaler, Julia Senkiv, Walter Jäger, Christian R. Kowol, Petra Heffeter, Walter Berger

**Affiliations:** ^1^ Department of Medicine I, Medical University of Vienna Institute of Cancer Research and Comprehensive Cancer Center Vienna Austria; ^2^ Research Cluster “Translational Cancer Therapy Research”, University of Vienna Vienna Austria; ^3^ Institute of Inorganic Chemistry, Faculty of Chemistry University of Vienna Vienna Austria; ^4^ Center for Pathobiochemistry and Genetics, Medical University of Vienna Vienna Austria; ^5^ University of Applied Sciences Upper Austria Wels Austria; ^6^ Department of Analytical Chemistry, Faculty of Chemistry University of Vienna Vienna Austria; ^7^ Department of Pediatrics and Adolescent Medicine Medical University of Vienna Vienna Austria; ^8^ Institute of Cell Biology NAS of Ukraine Lviv Ukraine; ^9^ Department of Pharmaceutical Chemistry, Division of Clinical Pharmacy and Diagnostics University of Vienna Vienna Austria

**Keywords:** Ponatinib, lipid droplets, resistance, cancer, drug sequestration

## Abstract

Ponatinib is a small molecule multi‐tyrosine kinase inhibitor clinically approved for anticancer therapy. Molecular mechanisms by which cancer cells develop resistance against ponatinib are currently poorly understood. Likewise, intracellular drug dynamics, as well as potential microenvironmental factors affecting the activity of this compound are unknown. Cell/molecular biological and analytical chemistry methods were applied to investigate uptake kinetics/subcellular distribution, the role of lipid droplets (LDs) and lipoid microenvironment compartments in responsiveness of FGFR1‐driven lung cancer cells toward ponatinib. Selection of lung cancer cells for acquired ponatinib resistance resulted in elevated intracellular lipid levels. Uncovering intrinsic ponatinib fluorescence enabled dissection of drug uptake/retention kinetics *in vitro* as well as in mouse tissue cryosections, and revealed selective drug accumulation in LDs of cancer cells. Pharmacological LD upmodulation or downmodulation indicated that the extent of LD formation and consequent ponatinib incorporation negatively correlated with anticancer drug efficacy. Co‐culturing with adipocytes decreased ponatinib levels and fostered survival of cancer cells. Ponatinib‐selected cancer cells exhibited increased LD levels and enhanced ponatinib deposition into this organelle. Our findings demonstrate intracellular deposition of the clinically approved anticancer compound ponatinib into LDs. Furthermore, increased LD biogenesis was identified as adaptive cancer cell‐defense mechanism *via* direct drug scavenging. Together, this suggests that LDs represent an underestimated organelle influencing intracellular pharmacokinetics and activity of anticancer tyrosine kinase inhibitors. Targeting LD integrity might constitute a strategy to enhance the activity not only of ponatinib, but also other clinically approved, lipophilic anticancer therapeutics.

## Abstract 2

**What's new?**

Ponatinib is a small‐molecule multi‐tyrosine kinase inhibitor clinically approved for anticancer therapy. However, to date, the intracellular pharmacokinetics of this compound and the molecular mechanisms underlying resistance in cancer cells remain largely unknown. Here, the authors found that ponatinib was selectively scavenged by lipid droplets in cancer cells. Ponatinib accumulation into lipid droplets emerged as a critical determinant of intrinsic and acquired drug resistance. The findings suggest that lipid droplets represent an underestimated organelle influencing intracellular pharmacokinetics and anticancer tyrosine kinase inhibitor activity. Moreover, co‐targeting of lipogenic cancer cell phenotypes might enhance the efficacy of ponatinib and other lipophilic pharmaceuticals.

**Abbreviations**
aCGH: array comparative genomic hybridization
ALL: acute lymphoblastic leukemia
ApoB/E: apolipoprotein B/E
CML: chronic myelogenous leukemia
FDR: false discovery rate
FGFR: fibroblast growth factor receptor
GO: gene ontology
GSEA: gene set enrichment analysis
LD: lipid droplet
MCC: Mander's Correlation Coefficient
OA: oleic acid
TC: triacsin C
TKI: tyrosine kinase inhibitor

## Introduction

Fibroblast growth factor receptors (FGFR) are a family of receptor tyrosine kinases serving as key regulators of manifold biological processes.^1^ As such, FGFR signaling is imperative in embryonic development as well as in the homeostasis of adult tissues.^2^, ^3^ With the advent of molecularly targeted therapy, FGFR aberrations have emerged as key tumorigenic factors in multiple cancer types.^1^, ^4^ These alterations include receptor mutation, translocation or amplification and are found with varying frequencies in carcinomas of the lung, breast, prostate, bladder, head and neck as well as in rhabdomyosarcoma.^5^, ^6^ Ponatinib is a small molecule multi‐tyrosine kinase inhibitor (TKI) approved for Bcr‐Abl‐rearranged, imatinib‐refractory chronic myelogenous and acute lymphoblastic leukemia (CML and ALL, respectively).^7^ Furthermore, ponatinib is currently being evaluated for clinical efficacy in multiple other cancer types including *FGFR1‐*amplified lung cancer.^8^ However, to date, molecular resistance mechanisms in cancer cells against this compound are widely unknown.

Lipid droplets (LDs, also termed adiposomes) are intracellular organelles present in virtually all mammalian cell types.^9^, ^10^ LDs are covered by an amphiphilic phospholipid monolayer which encloses a lipophilic core consisting mainly of triacylglycerides and cholesterol esters.^10^ Initially, these organelles were believed to represent a mere storage reservoir for excess intracellular lipids.^11^ In recent years, more detailed characterization of LD composition and regulation has uncovered that this organelle type is a key player at the crossroads of energy homeostasis and membrane biology as well as of the production of inflammatory markers.^12^ In cancer cells, LDs are often present in excessive amounts.^13^ This is believed to be the result of a lipogenic phenotype inherent to proliferative malignant cells to satisfy the high demand of metabolic fuel and building blocks for membrane biosynthesis.^14^ Moreover, LDs were suggested to represent accumulation sites for lipophilic substances such as vitamins or xenobiotics.^15^ Several studies suggested that an altered lipid metabolism of cancer cells has an impact on chemosensitivity, for instance by altered composition of plasma or mitochondrial membrane composition.^16^, ^17^ The role of LDs in that respect, however, is poorly understood.

In our study, we uncovered that a lipogenic switch underlies acquired ponatinib resistance of FGFR1‐driven lung cancer cells. By the newly identified intrinsic fluorescence activity of ponatinib, we discovered rapid and selective drug sequestration into intracellular LDs. To the best of our knowledge, these findings for the first time demonstrate direct accumulation of a pharmacological compound into this organelle. Short‐term as well as long‐term drug exposure resulted in increased LD load in cancer cells, going hand in hand with increased ponatinib deposition into this organelle. Analogously, LD enrichment of lung cancer cells by oleic acid (OA) supplementation potently reduced ponatinib activity, while LD depletion by the long‐chain fatty acyl‐CoA synthetase inhibitor triacsin C (TC) enhanced the killing potential of this TKI. Furthermore, we uncovered that the presence of a “lipoid” compartment *in vitro* distinctly diminished the anticancer activity of ponatinib in lung cancer cells, directly pointing toward a role of adipose tissue in humans as critical pharmacokinetic determinant of treatment efficacy.

In summary, our study demonstrates selective accumulation of an anticancer compound in LDs of cancer cells. LD tropism likely reflects the highly lipophilic nature of ponatinib. These findings highlight that the role of cell organelles in subcellular drug distribution and their influence on drug efficacy or failure are often poorly understood, even in case of clinically approved anticancer pharmaceuticals. Consequently, the intracellular behavior of anticancer compounds needs to be elucidated in greater detail in order to develop more efficient treatment modalities, for instance through rationale drug combinations that concomitantly target resistance‐conferring cancer cell phenotypes.

## Materials and Methods

In addition to the materials and experimental procedures described below, a detailed description of all remaining materials and methods used in this research article can be found in Supporting Information Materials and Methods.

### Materials

Ponatinib was purchased from Selleckchem (Munich, Germany). LysoTracker® Red and Bodipy 493/503 were obtained from Thermo Fisher Scientific (Waltham, MA). OA (bovine‐serum albumin (BSA)‐conjugated), dexamethasone, 3‐isobutyl‐1‐methylxanthine and insulin were purchased from Sigma (St. Louis, MO). TC was obtained from Cayman Chemical (Ann Arbor, MI). FGF‐basic (bFGF) was obtained from Peprotech (Rocky Hill, NJ).

### Array comparative genomic hybridization

4x44K oligonucleotide microarrays (Agilent, Santa Clara, CA) were used for direct array comparative genomic hybridization (aCGH) as described previously to compare indicated cell lines to normal human reference DNA as published.^18^ Labeling and hybridization of genomic DNA was performed according to the manufacturer's recommendations.

### Cell culture

The human lung cancer cell lines NCI‐H1703 (RRID: CVCL_1490), DMS114 (RRID:CVCL_1174) and A549 (RRID:CVCL_0023), as well as the CML cell line K562 (RRID:CVCL_0004) were obtained from American Type Culture Collection (ATCC, Manassas, VA). All cell lines were cultured in RPMI‐1640, supplemented with 10% fetal calf serum (FCS, PAA, Linz, Austria) at 5% CO_2_ and 37°C. To generate a ponatinib‐selected NCI‐H1703 and DMS114 subline, cells were exposed to low drug doses in regular intervals, followed by a drug‐free recovery phase. This procedure was applied over several months. All experiments including ponatinib‐selected sublines were performed with cells that were kept in drug‐free medium for at least 2 weeks. All human cell lines and their drug‐selected derivatives have been authenticated using short tandem repeat (STR) profiling within the last 3 years and all experiments were performed with *Mycoplasma*‐free cells (Mycoplasma Stain kit, Sigma). Red‐fluorescent variants of NCI‐H1703 cells were generated by transfection with pQCXIP‐mCherry‐IRES‐Puro plasmid DNA encoding mCherry and exhibiting an internal ribosomal entry site for a puromycin resistance gene. Then, 1 × 10^6^ cells were transfected with 5 μg plasmid DNA and subsequently selected using 0.75 μg/ml puromycin. Percentage of mCherry‐positive cells was checked constantly by flow cytometry. 3T3‐L1 mouse embryonic fibroblasts (designated here as 3T3‐L1/F) were purchased from ATCC (RRID:CVCL_0123). Differentiation of 3T3‐L1/F cells into an adipocytic phenotype (termed here 3T3‐L1/A) was performed by incubating cells in DMEM containing 7.6 μM dexamethasone, 200 μM 3‐isobutyl‐1‐methylxanthine and 1 μg/ml insulin (differentiation media) for 48 hr followed by 6 days incubation in post differentiation media containing 1 μg/ml insulin. Extent of differentiation was checked continuously by monitoring lipid accumulation in brightfield microscopy.

### Cell viability assay

Cells were pretreated with 100 μM OA, with 2 μM or with the indicated concentrations of TC for 72 hr. Subsequently, 3–5 × 10^3^ cells were plated in 96‐well plates and allowed to adhere overnight. For hypoxia experiments, cells were plated in 96‐well plates and preincubated at 0.1% O_2_ for 24 hr, followed by drug exposure for 72 hr (also at 0.1% O_2_). Cells were exposed to indicated concentrations of ponatinib for 72 hr. Cell viability was determined using the 3‐(4,5‐dimethylthiazol‐2‐yl)‐2,5‐diphenyltetrazolium bromide (MTT)‐vitality assay (EZ4U, Biomedica, Vienna, Austria). Data were analyzed using GraphPad Prism software (La Jolla, CA). IC_50_ values, indicating 50% reduced viability as compared to the untreated control were calculated by nonlinear regression curve‐fitting (sigmoidal dose–response with variable slope). Calcu Syn software (Biosoft, Ferguson, MO) was applied to evaluate synergistic effects of drug combinations.^19^ Drug effects are expressed as combination indices (CI), where values <0.9, 0.9–1.1 and >1.1 indicate synergism, additivity and antagonism, respectively.

### Whole‐genome gene expression array

Gene expression analysis was conducted using 4x44K oligonucleotide gene expression arrays (Agilent, Santa Clara, CA) as described previously.^20^, ^21^ Raw intensity values were read into R, background‐corrected and normalized using the normexp and loess algorithm of limma.^22^ Differentially perturbed gene ontology (GO) terms of the Kyoto Encyclopedia of Genes and Genomes (KEGG) and the Reactome databases were detected by gene set enrichment analysis (GSEA), choosing a maximum false discovery rate (FDR) cut‐off of <0.25 (http://www.broadinstitute.org/gsea/msigdb/index.jsp).

### Data and statistical analysis

The data and statistical analysis comply with the recommendations on experimental design and analysis in pharmacology.^23^ Data were analyzed using GraphPad Prism 6.0 software and are expressed as mean ± deviation (SD). If not stated otherwise, one out of at least three independent experiments, performed in triplicates, is depicted. Each data point represents the mean ± standard deviation SD derived from biological triplicate values, unless stated otherwise in the figure legends. One‐way or two‐way analysis of variance (ANOVA) with Bonferroni posttest as well as two‐tailed Student's *t*‐test or D'Agostino and Pearson omnibus normality test to check for Gaussian distribution followed by Mann–Whitney test were performed for statistical evaluation. *F* values and degrees of freedom (DF) for ANOVA, as well as *t* values and degrees of freedom for *t*‐test are indicated in respective figure legends. *p*‐values <0.05 were considered statistically significant. *p < 0.05; **p < 0.01; ***p < 0.001.

### Data availability

The data that support the findings of our study are openly available at ArrayExpress (https://www.ebi.ac.uk/arrayexpress/) under the accession number E‐MTAB‐8773.

## Results

### Selection for ponatinib resistance is accompanied by lipid metabolic reprogramming of FGFR‐driven lung cancer cells

**Figure 1:**
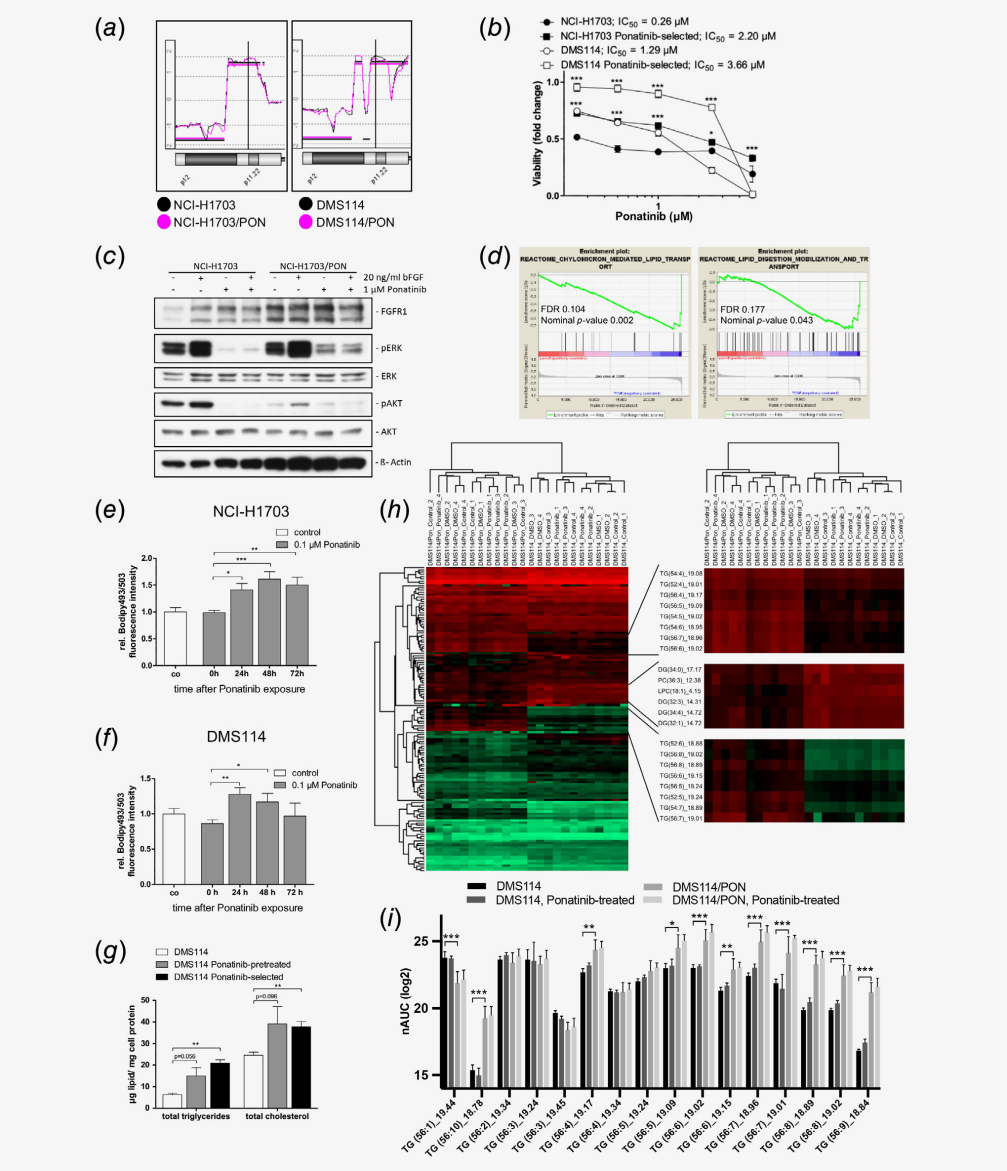
Acquired ponatinib resistance of FGFR1‐driven lung cancer cells is accompanied by lipid metabolic reprogramming. (*a*) Genome‐wide relative gene dose alterations of parental NCI‐H1703 and DMS114 cells as well as respective ponatinib‐selected sublines in comparison to normal diploid DNA were determined by aCGH. For each cell line, the respective chromosomal region harboring the FGFR1 locus at position 8p12 is depicted. (*b*) Viability of treatment‐naïve as compared to ponatinib‐selected NCI‐H1703 and DMS114 cells upon 72 hr treatment with increasing ponatinib concentrations was determined by MTT assay. *p < 0.05, ***p < 0.001, two‐way ANOVA, Bonferroni posttest. Asterisks are given above ponatinib‐selected cells at respective drug concentrations and indicate levels of significant difference in comparison to corresponding treatment‐naïve counterparts. *F* = 249.93, DF_group_ = 3, DF_residual_ = 48; (*c*) Phosphorylation levels of FGFR downstream effectors in parental and drug‐selected NCI‐H1703 cells upon exposure to 20 ng/ml bFGF for 5 min or to 1 μM ponatinib for 1 hr. β‐Actin served as loading control. (*d)*. Enrichment of lipid homeostasis‐associated genes differentially expressed in ponatinib‐selected as compared to treatment‐naïve DMS114 cells was determined by GSEA of genome‐wide mRNA expression analysis. Lipid‐associated, significant enrichment plots with a FDR cut‐off <0.25 of GO terms derived from the Reactome database are depicted. (*e*, *f*) The LD load of NCI‐H1703 (*e*) and DMS114 (*f*) cells in response to ponatinib exposure was determined by flow cytometry. Cells were treated for 1 hr with 1 μM ponatinib, followed by incubation in drug‐free medium. The cellular LD load was analyzed by Bodipy 493/503 incorporation at the indicated time points. Values are shown relative to the untreated control. *p < 0.05, **p < 0.01, ***p < 0.001, one‐way ANOVA, Bonferroni posttest. (*e*) *F* = 15.53, DF_group_ = 3, DF_residual_ = 8; (*f*) *F* = 7.101, DF_group_ = 3, DF_residual_ = 8; (*g*) Total triglyceride and cholesterol concentrations in ponatinib‐naïve, ‐pretreated, and ‐selected DMS114 cells were determined by gas chromatography. **p < 0.01, two‐ways Student's *t*‐test. Unselected *versus* pon‐selected: Triglycerides: *t* = 11.53, DF = 3; cholesterol: *t* = 6.638, DF = 3; (*h*) (*left*) Unsupervised hierarchical clustering of 135 lipid species identified in the lipidomic analysis of untreated and ponatinib‐treated DMS114 and DMS114/PON cells. (*right*) Magnification of indicated regions exhibiting pronounced shifts of lipid species between DMS114 and DMS114/PON cells. Upregulated lipids are shown in *red*, downregulated lipids are shown in *green*. In each row, lipid chain lengths and saturation are given in brackets besides corresponding lipid species. For details, see Supporting Information Materials and Methods. TG, triacylglyceride; DG, diacylglyceride; PC, phosphatidylcholine; LPC, lysophosphatidylcholine; (*i*) Bar charts of 16 identified triglycerides (TGs) and their normalized intensities, including their total number of carbon atoms in the three fatty acid chains and number of double bonds in parentheses, as well as their retention times in minutes. The standard deviations represent four biological replicates (duplicates of duplicates). Statistical significance levels of differences between lipid levels of untreated DMS114 as compared to DMS114/PON cells are depicted. *p < 0.05, **p < 0.01, ***p < 0.001, two‐way ANOVA, Bonferroni posttest. [Color figure can be viewed at wileyonlinelibrary.com]

Our study aimed at characterizing the molecular mechanisms underlying acquired unresponsiveness of cancer cells to ponatinib. To this end, the lung cancer cell lines NCI‐H1703 and DMS114 were employed as model system. These cell lines are driven by FGFR1 as a consequence of gene amplification on chromosome 8q (Fig. 1 *a*). Ponatinib‐resistant sublines of NCI‐H1703 and DMS114 cells were generated by constant exposure to low drug doses for several months. Despite retaining *FGFR1* copy number gains (Fig. 1 *a*), drug‐selected sublines were characterized by marked unresponsiveness toward ponatinib (Fig. 1 *b*). Both ponatinib‐selected cell lines retained FGFR signaling activity as illustrated by enhanced ERK and AKT phosphorylation upon stimulation with the potent FGFR1 ligand bFGF, while inhibition of these signaling modules was less potent as compared to parental cell lines (Fig. 1 *c*, Supporting Information Fig. S1 *a*). To further dissect transcriptional alterations in ponatinib‐selected cells, we performed genome‐wide mRNA expression microarray analysis of treatment‐naïve *versus* ponatinib‐selected DMS114 cells. Integrative bioinformatics suggested distinct perturbations in lipid‐metabolic processes. GSEA revealed enrichment of several GO terms within the Reactome and the KEGG databases concerning lipid metabolism. Significantly enriched gene sets in the ponatinib‐selected cell line comprised for instance “chylomicron‐mediated lipid transport” as well as “lipid digestion, mobilization and transport” (Fig. 1 *d*, Supporting Information Tables S1 and S2). Other enriched gene sets (although not meeting the false discovery rate (FDR) cut‐off) included “fatty acid metabolism” (nominal *p*‐value 0.000; FDR 0.255) and “lipid homeostasis” (nominal *p*‐value 0.051; FDR 0.377; data not shown). Within these GO terms, several genes directly or indirectly implicated in lipid homeostasis and lipoprotein assembly were deregulated. Consequently, we were interested whether ponatinib‐resistant cells might exhibit elevated intracellular lipid levels. To investigate this, we first employed flow cytometry to verify the feasibility to sensitively detect potential alterations in cellular lipid content, using Bodipy 493/503 as specific marker^24^, ^25^ (Supporting Information Fig. S1 *b*). Subsequently, NCI‐H1703 and DMS114 cells were treated with a single dose of ponatinib (0.1 μM) for 1 hr, followed by incubation in drug‐free media. Corroborating our hypothesis, Bodipy 493/503‐staining was significantly increased 24–72 hr after ponatinib exposure as compared to prior treatment (Fig. 1 *e*). In DMS114 cells, the same effect was observed although slightly less persistent as compared to the NCI‐H1703 model (Fig. 1 *f*).

Furthermore, we measured intracellular lipid levels in parental and ponatinib‐selected cancer cells by gas chromatography. Significantly elevated triglyceride as well as total cholesterol levels were observed in ponatinib‐selected DMS114 cells (Fig. 1g, Supporting Information Table S3). Also in the case of NCI‐H1703 cells, triglyceride levels were significantly increased in the drug‐selected subline (Supporting Information Fig. S1 *c* and Table S3). As already suggested by the data shown in Fig. 1 *f*, pretreatment of parental DMS114 cells with only one single, subtoxic dose of ponatinib resulted in markedly elevated triglyceride and cholesterol levels (Fig. 1g, Supporting Information Table S3). In addition, we performed mass spectrometry‐based lipidomic analysis to investigate the effect of ponatinib treatment on the lipid composition of DMS114 and DMS114/PON cells in further detail. Principal component analysis (PCA) using 135 identified lipid species revealed that sensitive and ponatinib‐selected phenotypes of DMS114 cells separated into distinct clusters (Supporting Information Fig. S1 *d*). In line with Fig. 1 *g*, PCA also suggested that exposure of parental DMS114 cells to a single dose of ponatinib moderately shifted the lipidomic signature toward that of DMS114/PON cells. Unsupervised clustering revealed a signature of upregulated triglycerides and downregulated diglycerides in DMS114/PON as compared to parental DMS114 cells (Fig. 1 *h*). Interestingly, DMS114/PON cells were characterized by an increased amount of (highly) polyunsaturated triglycerides compared to DMS114, while an inverse trend was observed for triglycerides and diglycerides with low numbers of double bonds (Fig. 1 *i*, Supporting Information Figs. S1 *e* and S1 *f*). Overall, these data point to a role of lipid metabolic reprogramming as adaptive cancer cell response toward ponatinib.

### Intrinsic fluorescence properties allow ponatinib detection *in vitro* and *in vivo*

**Figure 2:**
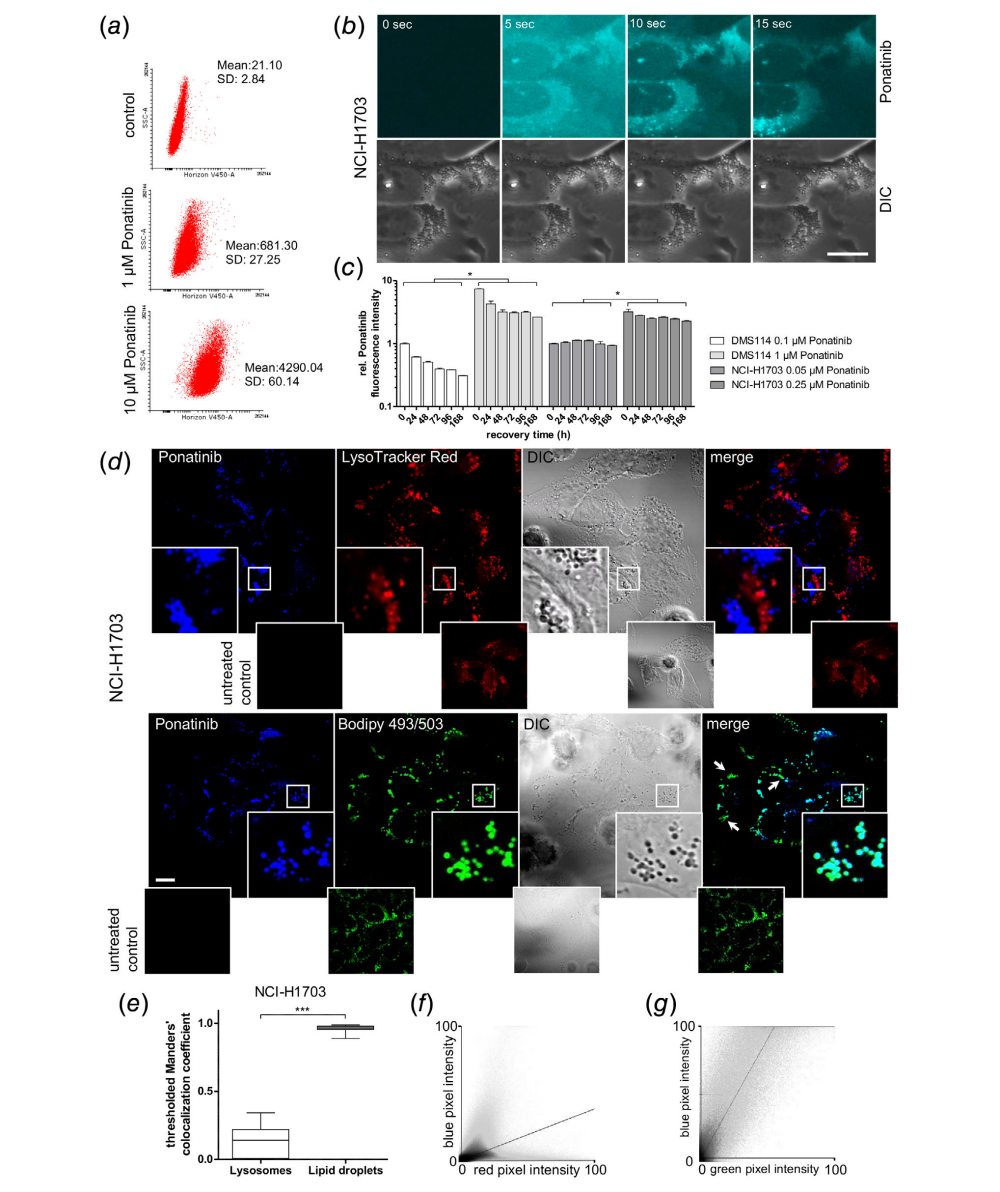
Intrinsic ponatinib fluorescence reveals selective accumulation in cancer cell LDs. (*a*) Ponatinib uptake in NCI‐H1703 cells was measured by flow cytometry after 1 hr exposure to the indicated drug concentrations. Ponatinib fluorescence emission was measured using the 405 nm laser and the 450/40 bandpass filter (Horizon V450 channel). (*b*) Short‐term uptake of 10 μM ponatinib in NCI‐H1703 cells was analyzed by live‐cell microscopy. Ponatinib is pseudo‐colored in cyan. The scale bar indicates 10 μm. (*c*) Ponatinib retention in DMS114 and NCI‐H1703 cells was analyzed by flow cytometry at the indicated time points. Cells were treated for 1 hr with the indicated concentrations of ponatinib, followed by incubation in drug‐free medium. Values were normalized to the 0 hr time points of the respective lower drug concentration for each cell line. One representative experiment, performed in biological triplicates, is shown out of three replicates. *F* = 2,402, DF_group_ = 5; DF_residual_ = 36; *p < 0.05, two‐way ANOVA, Bonferroni posttest. (*d*) Subcellular ponatinib localization in NCI‐H1703 cells was analyzed by confocal fluorescence microscopy. Cells were incubated with 10 μM ponatinib for 1 hr. LysoTracker® Red and Bodipy 493/503 served as markers for lysosomes and LDs, respectively. The scale bar indicates 10 μm. DIC, differential interference contrast; (*e*) Extent of colocalization of ponatinib‐ and lysosome/LD‐associated fluorescence signals was determined to calculate thresholded MCC. Single‐cell MCCs from at least three independent optical fields of three independent experiments were pooled (*n*_lysosome_ = 39, *n*_LD_ = 27). ***p < 0.001, D'Agostino and Pearson omnibus normality test, followed by two‐tailed Mann–Whitney test. (*f*, *g*) Pixel intensity correlations of ponatinib—Lysotracker® Red (*f*)/Bodipy 493/503 (G)‐derived signals from confocal micrographs (*d*). Representative scatter plots are shown. [Color figure can be viewed at wileyonlinelibrary.com]

Consequently, we hypothesized that an enhanced intracellular lipid content upon drug selection might impair ponatinib activity based on altered intracellular pharmacokinetics. Various anticancer compounds have been identified to be sequestered by lysosomes.^26^ This phenomenon, commonly referred to as lysosomotropism, is based on lipophilic and weakly basic physicochemical compound properties. This effect has been documented to reduce the activity of a number of prominent cytotoxic but also molecularly targeted anticancer agents including doxorubicin, gefitinib,^26^ and also nintedanib, another FGFR inhibitor.^27^ Hence, we set out to develop methods to analyze intracellular ponatinib distribution. Interestingly, we observed cell‐free ponatinib fluorescence by full‐range fluorescence spectroscopy, revealing a maximum emission at 468 nm at an excitation of 340 nm (Supporting Information Fig. S2 *a*). Importantly, drug fluorescence was retained *in vitro*, enabling the analysis of cellular ponatinib uptake by flow cytometry (Fig. 2 *a*) and fluorescence microscopy (Fig. 2 *b*). Intracellular ponatinib fluorescence intensity correlated with the administered dose. Additionally, ponatinib accumulation immediately reached a plateau phase at all investigated concentrations already from the first measurement point (5 min) onwards (Supporting Information Fig. S2 *b*). This suggests rapid saturation of intracellular drug levels *in vitro*. In fact, live‐cell microscopic imaging showed that strong accumulation in NCI‐H1703 cells occurred within seconds after drug exposure (Fig. 2 *b*). As ponatinib is clinically approved for the treatment of CML and ALL, we tested whether drug uptake is detectable also in the CML cell line K562. Indeed, K562 drug uptake reflected the data obtained from lung cancer cells (Supporting Information Fig. S2 *c*). Additionally, we were interested in intracellular retention kinetics of ponatinib. Live cell and flow cytometry washout experiments, in which cells were exposed to the drug for 1 hr followed by incubation in drug‐free media, revealed persistent intracellular presence of ponatinib for several days (Fig. 2 *c*, Supporting Information Fig. S2 *d*). These data demonstrate that the newly identified intrinsic fluorescence properties enable sensitive analysis of cellular ponatinib uptake and retention kinetics by fluorescence‐based techniques. Subsequently, we tested whether the intrinsic fluorescence properties make ponatinib amenable to direct imaging in organ and tumor tissue sections derived from orally treated mice. To this end, we established subcutaneous tumor xenografts of A549 lung cancer cells (as DMS114 and NCI‐1703 cells proved poorly tumorigenic in xenotransplantation models). Indeed, we found strong drug fluorescence in organs such as gastrointestinal tissue including the entire jejunal and ileal portions of the small intestine as well as, to a lesser extent, the colon (Supporting Information Figs. S3 *a*–S3 *d*). Importantly, ponatinib accumulation was also detectable in tumor tissue, albeit at lower intensity as compared to images derived from intestinal sections (Supporting Information Figs. S4 *a* and S4 *b*). These data support the feasibility of utilizing intrinsic fluorescence activity for detection of ponatinib in *in vivo* tissue cryosections to monitor important pharmacokinetic parameters such as dynamics of resorption by (or exposure of) gastrointestinal tissue, but also kinetics of intratumoral drug accumulation.

### Ponatinib selectively accumulates in LDs

Furthermore, we analyzed whether ponatinib might localize to lysosomes in cancer cells. However, confocal fluorescence microscopy of ponatinib‐treated NCI‐H1703 cells co‐stained with Lysotracker® Red did not suggest drug localization to lysosomes (Fig. 2 *d*, upper panel). Still, the focal appearance of ponatinib fluorescence suggested compartmentalization to a specific intracellular organelle. Strikingly, we found a strong colocalization of ponatinib‐ and Bodipy 493/503‐derived signals (Fig. 2 *d*, lower panel). Bodipy 493/503 is a lipophilic dye that selectively stains the cellular LD compartment.^24^ To evaluate the degree of spatial overlap, we correlated overlapping pixel intensities using thresholded Mander's Correlation Coefficient (MCC). This analysis yielded a low MCC (0.13) for ponatinib/Lysotracker® Red signals but a very high correlation coefficient (0.96) for ponatinib/Bodipy 493/503 signals (Fig. 2 *e*). Scatter plots showing drug/Lysotracker® Red and drug/Bodipy 493/503 pixel intensity correlations are depicted in Figures 2 *f* and 2*g*, respectively. These data point toward a role of LDs in acquired ponatinib resistance *via* direct drug scavenging.

### Cancer cell LD load impacts on intracellular ponatinib accumulation

**Figure 3:**
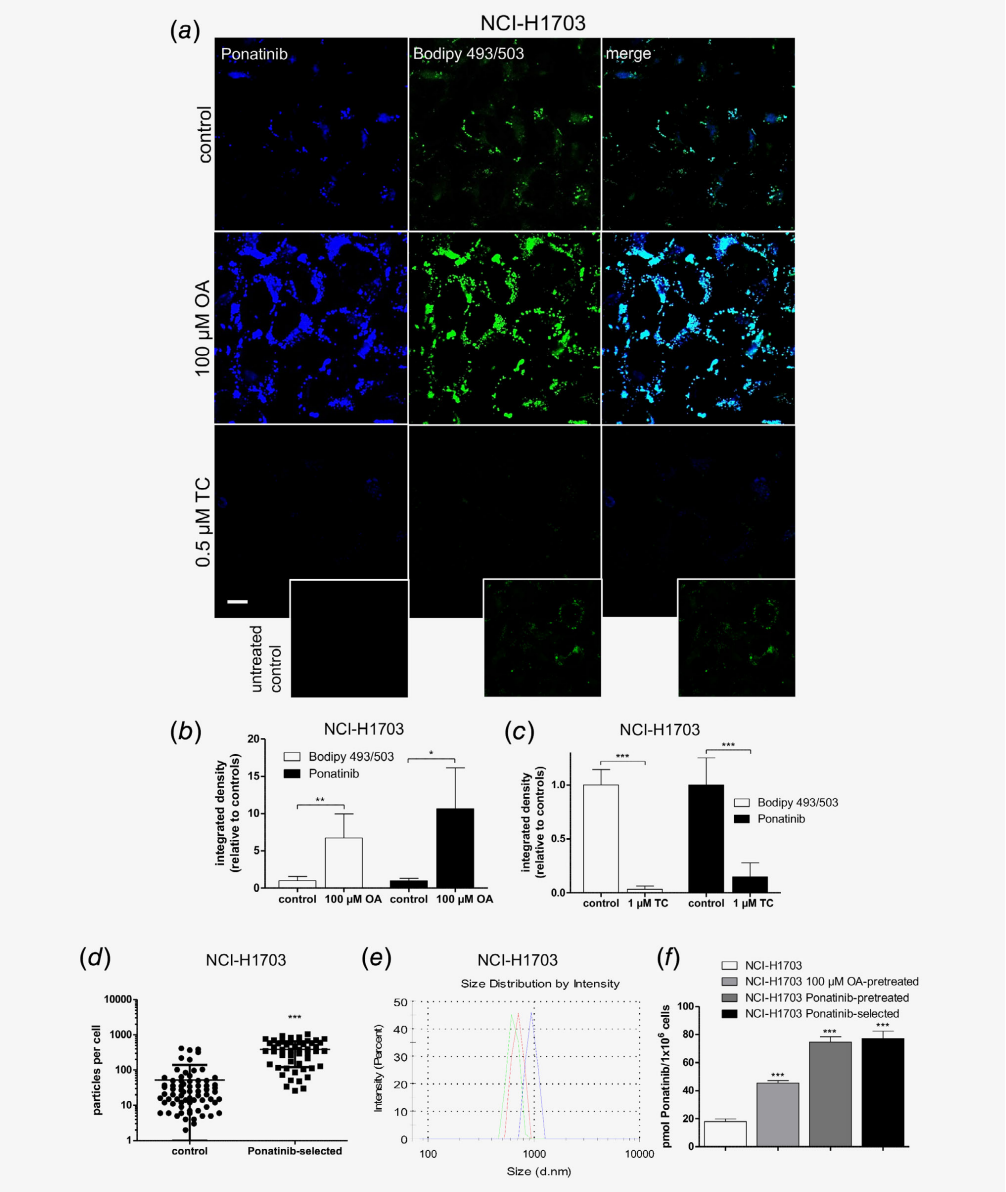
Modulation of the cellular LD load affects ponatinib incorporation. (*a*) Impact of LD enrichment and depletion by 72 hr pretreatment with 100 μM OA or 0.5 μM TC, respectively, on ponatinib incorporation in NCI‐H1703 cells was analyzed by confocal microscopy. Cells were treated for 1 hr with 10 μM ponatinib. Bodipy 493/503 served as LD stain. One representative out of at least three independent optical fields is depicted. The scale bar indicates 10 μm. (*b*, *c*) Quantification of ponatinib and Bodipy 493/503 fluorescence intensities from representative micrographs from at least three independent optical fields shown in (*a*) using ImageJ software. Values are shown relative to the ponatinib‐treated, OA/TC‐nonpretreated control. ***p < 0.001, two‐tailed Student's *t*‐test. Bodipy 493/503: *t* = 4.231, DF = 12; ponatinib: *t* = 3.035, DF = 4; (*d*) Impact of ponatinib‐selection on LD numbers was evaluated by quantification of confocal micrographs of Bodipy 493/503‐stained LDs in NCI‐H1703 cells using ImageJ software‐based particle analysis. For each experimental condition, a minimum of 10 individual cells from at least three independent micrographs were analyzed. (*n*_control_ = 71, *n*_ponatinib‐selected_ = 51) ***p < 0.001, D'Agostino and Pearson omnibus normality test, followed by two‐tailed Mann–Whitney test. (*e*) The size distribution of LDs isolated form NCI‐H1703 cells was determined by DLS. One representative experiment, performed in triplicates is shown. (*f*) Ponatinib concentrations were quantified by HPLC in isolated LDs of NCI‐H1703 cells that were (1) untreated, (2) 72 hr pretreated with 100 μM OA, (3) 1 hr pretreated with 0.1 μM ponatinib, followed by 72 hr recovery and (4) ponatinib‐selection. Before LD isolation, cells were treated with 10 μM ponatinib for 1 hr. ***p < 0.001, two‐tailed Student's *t*‐test. OA‐pretreated: *t* = 18.02, DF = 4; pon‐pretreated: *t* = 23.34, DF = 4; pon‐selected: *t* = 18.14, DF = 4. [Color figure can be viewed at wileyonlinelibrary.com]

As a next step, we were interested whether modulation of the intracellular LD pool might alter intracellular ponatinib distribution. We, thus, supplemented cell culture media with OA, a potent inducer of triacylglyceride synthesis and LD biogenesis. Corroborating our hypothesis, LD enrichment resulted in strongly increased intracellular ponatinib fluorescence in cells treated for 1 hr with 10 μM of the drug (Fig. 3 *a*). Moreover, LD formation inhibition by subtoxic concentrations of TC (*via* blocking long‐chain fatty acyl‐CoA synthetase‐mediated triglyceride and cholesterol ester formation) led to a markedly lower ponatinib deposition (Fig. 3 *a*, Supporting Information Fig. S5 *a*). Of note, total intracellular ponatinib concentrations were elevated by OA, but not decreased by TC treatment as compared to untreated controls (Supporting Information Fig. S5 *b*). Image analysis revealed that the significantly increased LD number (Supporting Information Fig. S5 *c*) and average size (Supporting Information Fig. S5 *d*) of OA‐supplemented cells went hand in hand with a significantly elevated, LD‐associated ponatinib fluorescence intensity (Fig. 3 *b*). Importantly, when treated with TC, the opposite was observed (Fig. 3 *c*, Supporting Information Figs. S5 *c* and S5 *d*).

Interestingly, also ponatinib‐selected NCI‐H1703 cells exhibited a significantly increased number of LDs as compared to treatment‐naïve cells (Fig. 3 *d*). We were, thus, interested in whether ponatinib selection leads to increased drug deposition into LDs. Hence, we isolated nascent LDs of OA‐treated, ponatinib‐single treated, as well as selected NCI‐H1703 cells. The average size distribution of LDs ranged between 711 and 958 nm (Fig. 3 *e*). Indeed, HPLC measurements demonstrated that OA‐mediated LD induction yielded significantly elevated ponatinib levels in the LD fraction as compared to nonpretreated cells (Fig. 3 *f*). Strikingly, ponatinib‐selected cells, as well as cells having received only a single subtoxic dose of ponatinib exhibited even higher LD‐associated drug levels than their OA‐supplemented counterpart (Fig. 3 *f*). This effect was accompanied by marked transcriptional upregulation of the LD markers *PLIN1*, *PLIN2* and *PLIN3* in DMS114 cells 72 hr after short‐term, single‐dose exposure to ponatinib (Supporting Information Fig. S5 *e*). The same effect was observed for *PLIN1* and *PLIN2*, but not of *PLIN3* in NCI‐H1703 cells (Supporting Information Fig. S5 *f*). Importantly, elevated protein levels of the LD markers ADRP (*PLIN2*) and TIP47 (*PLIN3*) were detected in DMS114/PON as compared to parental cells (Supporting Information Fig. S5 *g*).

As hypoxia has been reported to induce LD formation,^28^ we investigated the effect of hypoxia on cancer cell sensitivity toward ponatinib. However, drug treatment of DMS114 and NCI‐H1703 cells under hypoxic conditions did not result in desensitization against ponatinib (Supporting Information Fig. S5 *h* and S5 *i*). In addition, induction of ER stress was not apparent in DMS114 cells treated with ponatinib (Supporting Information Fig. S5 *j*).

In conclusion, our data provide strong indications that both short‐term and constant exposure of cancer cells toward ponatinib elicits an adaptive response fostering enhanced LD formation preceded by rapid drug accumulation in this organelle (compare Fig. 2 *b*). Our observations suggest a—to the best of our knowledge previously unprecedented—capacity of cancer cells to dynamically enhance the LD load upon drug contact, as a subcellular deposition strategy to limit its cytotoxic potential.

### The LD load influences cancer cell sensitivity toward ponatinib

**Figure 4:**
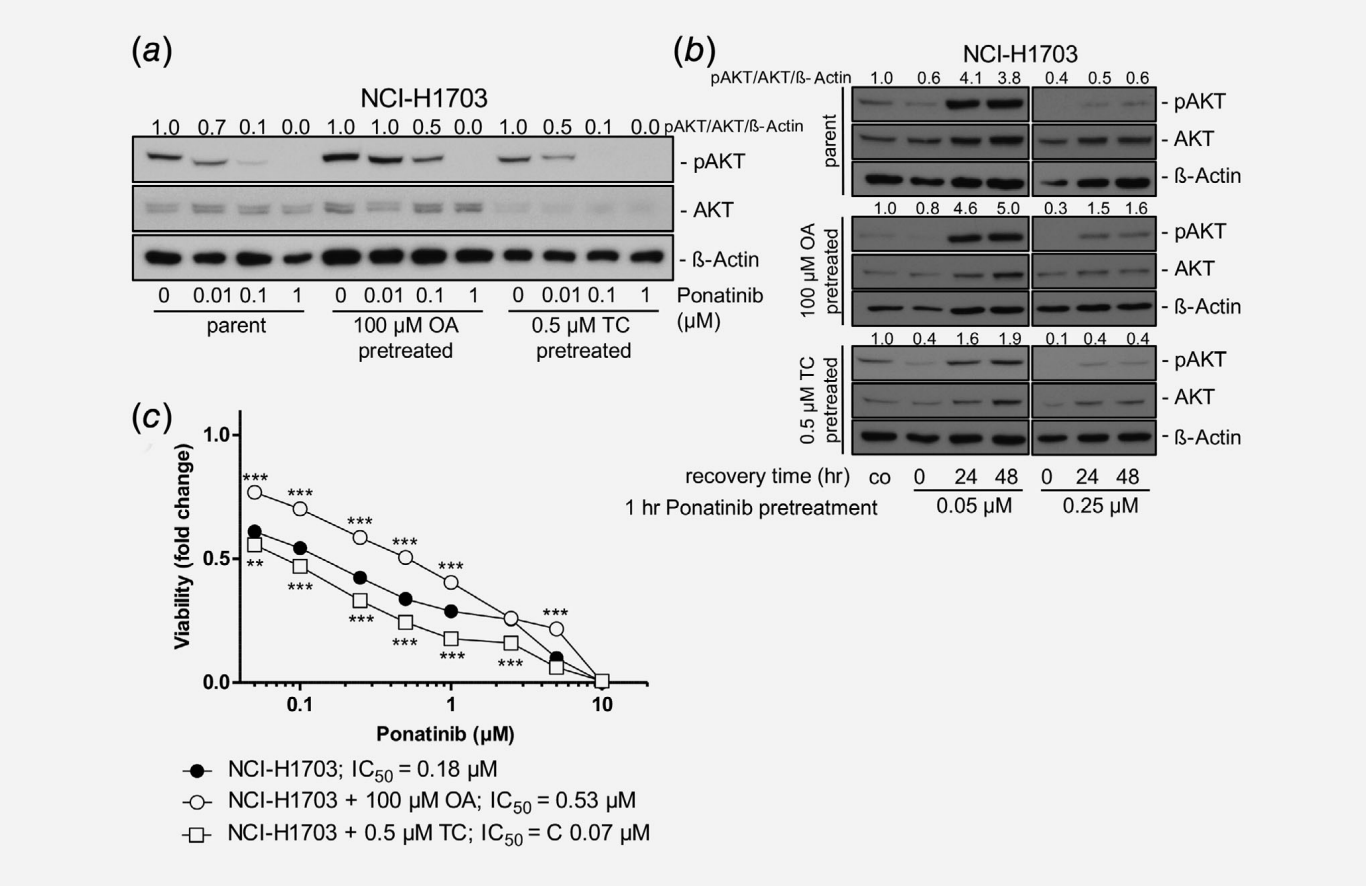
Modulation of intracellular lipid homeostasis impacts on the activity of ponatinib. (*a*) Impact of 72 hr pretreatment with 100 μM OA or 0.5 μM TC on the inhibitory potential of increasing ponatinib concentrations on FGFR signaling in NCI‐H1703 cells was analyzed by Western blot. β‐Actin served as loading control. Quantification of AKT phosphorylation levels is shown. Phosphorylation levels were normalized to total AKT and β‐Actin expression and are shown relative to respective ponatinib‐untreated controls. (*b*) Impact of treatment with 100 μM OA or 0.5 μM TC on FGFR downstream signaling, after an 1 hr preincubation of NCI‐H1703 cells with the indicated concentrations of ponatinib. Quantification of AKT phosphorylation levels is depicted for each experimental condition. Values were normalized to total AKT and β‐Actin expression and are shown relative to respective ponatinib‐untreated controls. (*c*) Impact of 72 hr preincubation with 100 μM OA or with indicated concentrations of TC on cell viability of NCI‐H1703 cells, treated for 72 hr with increasing concentrations of ponatinib was analyzed by MTT assay. Asterisks indicate levels of significance of difference at respective ponatinib concentrations between OA‐/TC‐pretreated and nonpretreated cells. *p < 0.05, **p < 0.01, ***p < 0.001, two‐way ANOVA, Bonferroni posttest. *F* = 378.33, DF_group_ = 2, DF_residual_ = 54.

Consequently, we aimed at analyzing whether LD‐mediated drug scavenging indeed plays a role in the sensitivity of the investigated cell lines toward ponatinib. Of note, LD enrichment in OA‐pretreated NCI‐H1703 cells led to a markedly decreased potential of ponatinib to inhibit FGFR downstream signaling. This was illustrated by more sustained AKT phosphorylation as compared to nonpretreated cells (Fig. 4 *a*). Importantly, TC pretreatment had the opposite effect, as AKT phosphorylation was almost completely abolished already upon incubation with 0.01 μM ponatinib (Fig. 4 *a*). In another experimental setting, NCI‐H1703 cells were treated for 1 hr with the indicated concentrations of ponatinib, followed by drug removal and incubation in media supplemented with either 100 μM OA or 0.5 μM TC. Here, recurrence of AKT phosphorylation levels was more pronounced in OA‐treated as compared to parental cells after both 24 and 48 hr (Fig. 4 *b*). In contrast, LD depletion by TC potentiated the inhibitory activity of ponatinib also in this experimental setting. Hence, rebound of AKT phosphorylation was distinctly weaker than that of cells grown in unsupplemented media (Fig. 4 *b*). Thus, LD modulation has an impact on the capacity of ponatinib to inhibit FGFR1 signaling. In line with this, we found that LD enrichment significantly decreased NCI‐H1703 cell sensitivity toward ponatinib (3.0‐fold increased IC_50_ value, respectively), whereas LD depletion led to markedly enhanced ponatinib activity (2.6‐fold decreased IC_50_ value, respectively; Fig. 4 *c*). Importantly, the same effect was observed in DMS114 cells (Supporting Information Fig. S6 *a*). Of note, TC pretreatment of NCI‐H1703/PON as well as DMS114/PON cells also resulted in enhanced ponatinib activity, although with slightly reduced efficacy as compared to respective parental models (Supporting Information Figs. S6 *b* and S6 *c*).

Overall, this provides evidence that LD‐mediated drug scavenging is an important determinant of cellular responsiveness toward ponatinib.

### Lipoid compartments reduce ponatinib availability and foster survival of treated cancer cells

**Figure 5:**
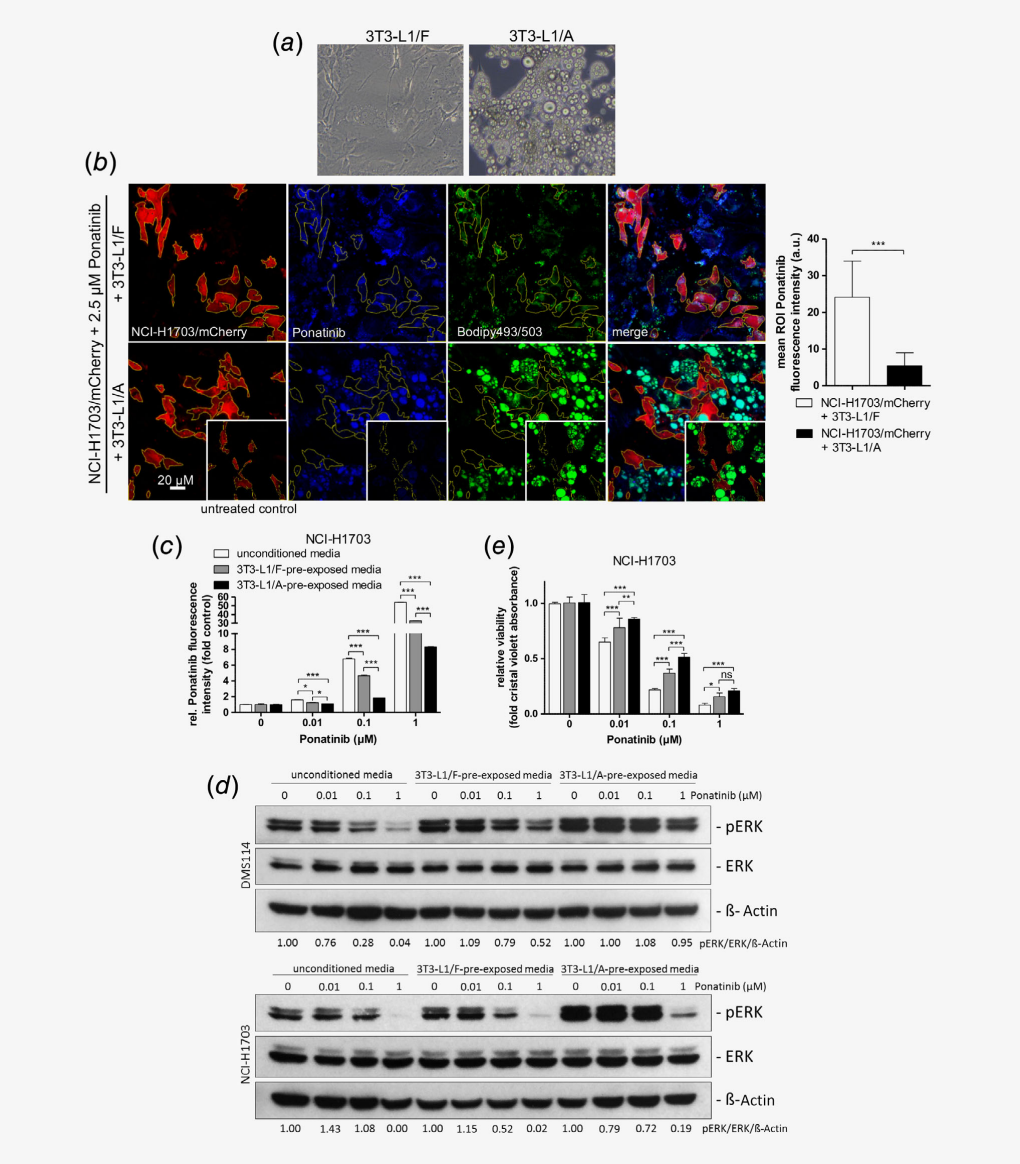
Adipocytes constitute a ponatinib‐scavenging reservoir and decrease its activity against adjacent cancer cells. (*a*) 3T3‐L1/A cells with adipocytic phenotype were generated by differentiation of 3T3‐L1/F fibroblasts. Representative brightfield images are depicted prior to and 23 days post differentiation. (*b*) Uptake of 2.5 μM ponatinib into NCI‐H1703/mCherry cells, treated for 1 hr in the presence of 3T3‐L1/F or 3T3‐L1/A cells was determined by confocal fluorescence microscopy. Cancer cells were delineated by creating ROI around mCherry‐positive areas using ImageJ software. Ponatinib was quantified by measuring blue pixel intensities in every single mCherry‐positive ROI, and by subtracting background fluorescence in ROI of respective untreated control images (*right panel*). Mean single‐cell ROI intensities of at least three independent images were pooled (*n*_3T3‐L1/F_ = 37, *n*_3T3‐L1/A_ = 34). ***p < 0.001, D'Agostino and Pearson omnibus normality test, followed by Mann–Whitney test. (*c*) Ponatinib accumulation in NCI‐H1703 cells was determined by flow cytometry. 3T3‐L1/F and 3T3‐L1/A cells were incubated for 24 hr with indicated concentrations of ponatinib. Supernatant was transferred onto adherent NCI‐H1703 cells and incubated for 1 hr, followed by flow cytometric analysis. Fluorescence intensities are depicted as values relative to the untreated control. One representative experiment, performed in triplicates, is depicted. ***p < 0.001, two‐way ANOVA, Bonferroni posttest. *F* = 58,676.17, DF_group_ = 2, DF_residual_ = 12; (*d*) The effect of 3T3‐L1/F and 3T3‐L1/A cell‐conditioning on the signaling‐inhibitory potential of ponatinib was determined by Western blot analysis. 3T3‐L1/F and 3T3‐L1/A cells were incubated for 24 hr with indicated concentrations of ponatinib. Supernatant was transferred onto adherent DMS114 and NCI‐H1703 cells and incubated for 1 hr. β‐Actin served as loading control. Quantification of ERK phosphorylation levels is shown below corresponding lanes. Phosphorylation levels were normalized to total ERK and β‐Actin expression and are shown relative to respective ponatinib‐untreated controls. (*e*) Impact of 3T3‐L1/F and 3T3‐L1/A cell‐conditioning on the anticancer activity of ponatinib was determined by clonogenic assay. 3T3‐L1/F and 3T3‐L1/A cells were incubated for 24 hr with indicated concentrations of ponatinib. Pre‐exposed, as well as non pre‐exposed supernatants, were transferred onto adherent NCI‐H1703 cells and incubated for 168 hr. Quantification of fixed, crystal violet‐stained cells are depicted. Values are normalized to respective untreated controls. **p < 0.01, ***p < 0.001, two‐way ANOVA, Bonferroni posttest. ns, nonsignificant. *F* = 88.62, DF_group_ = 2, DF_resiual_ = 60. [Color figure can be viewed at wileyonlinelibrary.com]

Based on the observation that ponatinib accumulates in LDs, we further wanted to investigate whether lipoid cell types might scavenge this drug and lower its concentrations available to target cancer cells. Some anticancer compounds are known to accumulate in lipophilic body compartments such as adipose tissue, strongly influencing their systemic pharmacokinetic properties.^29^ Hypothesizing that this effect also applies to ponatinib, we aimed at mimicking this interplay *in vitro*. To this end, we differentiated 3T3‐L1 fibroblasts (3T3‐L1/F) into an adipocytic phenotype (3T3‐L1/A; Fig. 5 *a*). Strikingly, co‐culture microscopy experiments of 3T3‐L1/A adipocytes with mCherry‐transfected NCI‐H1703 cells exhibited that ponatinib accumulation in cancer cells was massively decreased as compared co‐culturing with 3T3‐L1/F cells (Fig. 5 *b*). Furthermore, significantly decreased drug levels in cancer cells incubated with ponatinib‐containing supernatants pre‐exposed to 3T3‐L1/A as compared to both 3T3‐L1/F cells and non pre‐exposed drug solutions were confirmed by flow cytometry (Fig. 5 *c*). We, thus, investigated whether the decreased drug availability also results in decreased inhibitory potential of ponatinib on its target kinases (e.g., FGFR1) in cancer cells. Indeed, incubation of DMS114 and NCI‐H1703 cells with ponatinib‐containing supernatants revealed that its inhibitory potential was distinctly weaker in 3T3‐L1/A‐pre‐exposed, as compared to both non pre‐exposed, as well as 3T3‐L1/F‐pre‐exposed media, as indicated by sustained ERK phosphorylation (Fig. 5 *d*). Ultimately, we found that ponatinib‐scavenging by 3T3‐L1/A adipocytes significantly stronger reduced the anticancer potential of ponatinib as compared to 3T3‐L1/F fibroblasts (Fig. 5 *e*). This implies that 3T3‐L1/A cells exhibit a distinctly enhanced capacity to scavenge the lipophilic compound ponatinib compared to their isogenic fibroblast counterpart. In conclusion, these data strongly suggest that the presence of lipoid compartments in the body as well as in the tumor microenvironment distinctly impacts on ponatinib pharmacokinetics and its anticancer potential by constituting highly efficient storage reservoirs.

## Discussion

Subcellular distribution of cytotoxic agents as well as of modern targeted compounds and its impact on the respective anticancer activities needs to be better understood. Mathematical algorithms hold great potential in predicting these dynamics, but to date still encounter limitations due to the large complexity of the interplay between the physicochemical make‐up of pharmacological compounds and the biological as well as the biophysical properties of respective target cells.

LDs have long been considered as inert depots for the storage of excess intracellular lipids.^11^ More recently, this organelle has received increased attention, and its role as central hub between energy metabolism, membrane homeostasis and the production of inflammatory mediators is becoming increasingly recognized.^10^ In cancer cells, lipid metabolic reprogramming has emerged as key player, driving the supply of metabolic fuel and of building blocks needed for the production of membranous cell compartments.^14^ In line with this, excessive LD amounts have been documented in many cancer types, including those of the liver, breast and prostate.^13^ The LD status, comprising for example LD numbers or volumes, is positively correlated with malignancy in various reports. For instance, increased LD load is associated with enhanced aggressiveness and stemness of breast and colorectal carcinoma, respectively.^30^, ^31^ Of note, lipid reprogramming is also implicated in certain aspects of drug resistance. For instance, fatty acid synthase (FAS) expression levels exhibit an inverse correlation with sensitivity of breast cancer cells toward chemotherapy.^16^ In addition, alterations of the plasma or mitochondrial membrane composition by increased cholesterol levels have been associated with drug resistance.^17^ For these reasons, various pharmacological compounds have been developed to target the lipogenic phenotype of cancer cells. These comprise, among others, inhibitors of fatty acid metabolism (e.g., cerulenin, targeting FAS or TC, blocking long chain fatty acyl CoA synthetase) or of cholesterol synthesis (such as statins or avasimibe, which inhibits cholesterol esterification by blockade of sterol O‐acyltransferase 1).^32^

Several organelles serve as accumulation sites for anticancer compounds. Above all, lysosomes have been recognized to sequester a broad range of anticancer agents, many of which are in clinical use.^26^ This phenomenon is commonly believed to be caused by weakly basic physicochemical compound properties resulting in protonation in the acidic luminal environment and, thus, lysosomal trapping.^26^ In contrast, the potential role of LDs with respect to anticancer therapy resistance is poorly understood. Accordingly, hints pointing to a direct involvement of this organelle in drug resistance as intracellular drug scavengers are scarce. Two studies suggested LD accumulation of curcumin—a preclinically tested polyphenol—and docetaxel in glioblastoma and breast cancer cells, respectively.^15^, ^33^ Tamoxifen‐resistant breast cancer cells have recently been shown to induce lipid reprogramming, including hyper‐activation of cholesterol biosynthesis and deposition of neutral lipids in LDs.^34^ A recent mechanistic study showed that LD content and lysophosphatidylcholine acyltransferase 2 (LPCAT2) levels are positively correlated in colorectal carcinoma cells, and that LPCAT2‐mediated LD accumulation induced cancer cell resistance against 5‐fluorouracil and oxaliplatin.^35^ Furthermore, oncogene‐ablation refractory breast and pancreatic cancer cells were found to induce a metabolic shift associated with accumulation of LDs.^36^, ^37^ In addition, the histone deacetylase (HDAC) inhibitor entinostat, which undergoes clinical testing against several cancer types,^38^ induced LD accumulation in human hepatoma cells.^39^ Another preclinical isoquinoline compound, tetrazanbigen, was suggested to exert its cytotoxic effects explicitly *via* LD formation,^40^ arguing against therapeutic combination with agents that lose activity in a lipogenic cancer cell background. However, it cannot be completely ruled out that—analogously to ponatinib—LD induction by tetrazanbigen may depict a pharmacological bystander effect, caused by lipid metabolic reprogramming in response to this compound rather than being the actual cytotoxic mode‐of‐action. Importantly, the current consensus arising from this body of literature places lipid metabolic reprogramming as key mechanism maintaining energetic balance, as well as fostering cancer cell survival through altered membrane rigidity and sustained anti‐apoptotic signaling.

To the best of our knowledge, our study is the first to provide direct evidence that a clinically approved anticancer compound selectively accumulates in LDs. This compartmentalization effect represents a considerable factor limiting the inhibitory potential of this TKI. Furthermore, our study provides the additional and unprecedented finding that an adaptively increased LD load serves as actual deposition site for an anticancer compound, and that this effect fosters cancer cell survival, likely by reducing drug concentrations available for target kinase inhibition. This observation clearly points toward a role of increased LD biogenesis in the context of cancer cell reprogramming into a lipogenic phenotype as an adaptive mechanism, limiting the activity of lipophilic anticancer agents such as ponatinib *via* direct scavenging. Regarding this, it is worth questioning how the lipogenic switch elicited by the above‐mentioned compounds establishes drug resistance in cancer cells. It might be hypothesized that LD scavenging constitutes a more widely acting mechanism to reduce cytoplasmic drug concentrations. However, log*p* values of anticancer compounds that induce LD biogenesis vary strongly, with some exhibiting distinctly lower lipophilicity than ponatinib (log*p* = 4.32, pubchem.ncbi.nlm.nih.gov). On the one hand, it is likely that compounds with similar or higher log*p* values as compared to ponatinib (e.g., 3.00, 3.29 and 7.10 for paclitaxel, curcumin and tamoxifen, respectively) may indeed also accumulate in LDs. On the other hand, induction of these organelles by other, more water‐soluble agents (e.g., oxaliplatin, 5‐fluorouracil and docetaxel with log*p* values of −0.47, −0.89 and 2.40, respectively) probably mediates drug resistance *via* alternative lipid metabolism‐associated mechanisms. Furthermore, it has to be considered that compounds with lipophilicities considerably lower than ponatinib exhibit distinctly different intracellular pharmacokinetics. For instance, nintedanib (with a log*p* of 1.89 still considered more lipophilic than oxaliplatin and 5‐fluorouracil) is sequestered by lysosomes, not by LDs.^27^ This illustrates a multifaceted role of lipid reprogramming, and lipophilicity not as the sole physicochemical determinant of LD induction and deposition as cancer cell resistance mechanism.

It will be interesting to evaluate whether the combination of LD‐targeting agents with ponatinib exerts synergistic anticancer effects or reverses/prevents LD upregulation‐mediated resistance against this inhibitor. With regard to this, two pharmacological isoflavones, genistein and daidzein, were shown to disrupt LD formation, accompanied by downregulation of LD‐associated proteins such as Perilipin‐1, ADRP and Tip‐47.^41^ Furthermore, recently PFK158, an inhibitor which blocks the key glycolytic enzyme 6‐phosphofructo‐2‐kinase/fructose‐2,6‐biphosphatase 3, was found to induce lipophagy in gynecological cancer cells.^42^ Interestingly, this effect potentiated the anticancer activity with paclitaxel—the structural and functional analog of docetaxel—which was already demonstrated in independent studies to work less efficiently in breast cancer cells with a progestin‐induced increased LD load.^33^ It would, thus, be interesting to test in future studies whether pharmacologic inhibitors of glycolytic pathways/inducers of lipophagy might synergize also with ponatinib. In addition, the observed prolonged ponatinib retention in LDs raises the question about the intracellular pharmacokinetic fate of this drug. We hypothesize that one major driving mechanism reducing ponatinib concentration is determined by the proliferation rate of cells (exposed to only subtoxic ponatinib doses), leading to gradual drug dilution as cells divide. This is unlike biological responses described, for example, for lysosomotropic agents, for which activation of drug export *via* exocytic vesicles is observed already in the range of several hours after drug exposure.^43^

Ultimately, our data clearly demonstrate that lipoid cell compartments limit the killing potential of ponatinib toward cancer cells, which points to a critical role of adipose tissue in the pharmacokinetics and activity of ponatinib in human patients.

In conclusion, our study elucidates the impact of adipose tissue compartments on ponatinib efficacy and constitutes the basis to study intracellular drug dynamics in further detail. Moreover, our data demonstrate for the first time that a lipophilic anticancer compound accumulates in LDs, and that increased biogenesis of these organelles is exploited by cancer cells in frame of an adaptive response to hamper drug activity. Thus, these organelles, *via* direct scavenging, play a yet underestimated role with respect to their potential to influence intracellular pharmacokinetics and activity of lipophilic anticancer compounds such as ponatinib. It will be crucial in the future to elucidate whether pharmacologic targeting of the lipogenic phenotype or compromising of LD integrity go hand in hand with enhanced activity not only of ponatinib, but also of other clinically‐approved anticancer agents.

## Conflict of interest

The authors declare no conflicts of interest.

## Supporting information


**Appendix S1**: Supporting Information
