# Supplementary material for: Lipid droplet‐mediated scavenging as novel intrinsic and adaptive resistance factor against the multikinase inhibitor ponatinib
Source: Int J Cancer. 2020 Mar 2;147(6):1680–93. doi: 10.1002/ijc.32924 (PMC7497038; doi:10.1002/ijc.32924)
Supplement: Supplementary file 1 — Appendix S1: Supporting Information [file IJC-147-1680-s001.pdf]

SUPPLEMENTARY MATERIAL

LIPID DROPLET-MEDIATED SCAVENGING AS NOVEL INTRINSIC AND ADAPTIVE RESISTANCE FACTOR AGAINST THE MULTIKINASE INHIBITOR PONATINIB

Bernhard Englinger, Anna Laemmerer, Patrick Moser, Sebastian Kallus, Clemens Röhrl, Christine Pirker, Dina Baier, Thomas Mohr, Laura Niederstaetter, Samuel M. Meier-Menches, Christopher Gerner, Lisa Gabler, Johannes Gojo, Gerald Timelthaler, Julia Senkiv, Walter Jäger, Christian R. Kowol, Petra Heffeter, Walter Berger.

Table of contents

Supplementary materials and methods……………………………………………………2

Table S1………………………………………………………………………………13

Table S2………………………………………………………………………………13

Table S3………………………………………………………………………………14

Figure S1……………………………………………………………………………16

Figure S2……………………………………………………………………………19

Figure S3……………………………………………………………………………21

Figure S4……………………………………………………………………………23

Figure S5……………………………………………………………………………25

Figure S6……………………………………………………………………………27

References……………………………………………………………………………28

Supplementary materials and methods

Fluorescence spectroscopy

The 3-dimensional fluorescence spectrum of ponatinib was determined using a Horiba FluoroMax®-4 spectrofluorometer (Kyoto, Japan). Data were analyzed using FluorEssence v3.5 software. Dimethylsulfoxide (DMSO) stock solutions of ponatinib were diluted with phosphate-buffered saline (PBS) (10 mM, pH 7.4) to 15 µM (final DMSO concentration 1%). Fluorescence spectra were determined at excitation wavelengths from 220 nm to 420 nm and emission was recorded within the range of 240-700 nm at room temperature with excitation and emission slit widths of 5 nm.

Imaging of ponatinib in tissue cryosections

To detect ponatinib in cryosections of organ and tumor tissue of mice, $5x10^5$ A549 lung cancer cells were injected subcutaneously into the right flank of four 8 weeks old, male SCID mice, each weighing 20 g. Animals were obtained from Charles River Laboratories (Wilmington, MA, USA). Animal experiments were authorized by the Ethics committee at the Medical University of Vienna and carried out in accordance with the guidelines for the welfare and use of animals in cancer research, as well as meeting the Federation of Laboratory Animal Science Associations (FELASA) guidelines' definition of humane endpoints and the Arrive guidelines for animal care and protection, also strongly considering the strategies to replace, reduce, and refine ("3R")[1]. Upon tumor formation, two mice received a single oral dose of 30 mg ponatinib per kg bodyweight dissolved in ddH$_2$O containing 10% Chremophor (Sigma), 10% DMSO and 5% ethanol (Sigma) 46 days post-engraftment. Two mice received solvent only. Two hours after administration, mice

were sacrificed and organ and tumor tissue was embedded and frozen in OCT medium (Sakura Finetek, Staufen, Germany). 10 µm sections were sliced on a cryomicrotome (Thermo Fisher Scientific), fixed with 4% PFA on Superfrost Plus microscope slides (Thermo Fisher Scientific) and counterstained with 5 µg/ml propidium iodide (PI, Sigma) for 30 minutes at 4°C following treatment with 100 µg/ml RNAse A (Sigma) for 30 minutes at 37°C. To visualize histology as well as live/dead cells, consecutive tissue sections were stained with hematoxylin and eosin (H&E, Sigma). Slides were digitized using a Pannoramic Midi Slide Scanner (3DHISTECH, Budapest, Hungary) and Pannoramic Viewer software (3DHISTECH). Matching fluorescence images (8-bit integers, grayscale values ranging from 0 to 255) were acquired on a Zeiss LSM700 confocal laser scanning microscope using a 63x oil immersion objective. Image analysis was performed using Tissue Studio software (Definiens, Munich, Germany). In each tissue slice of all mice, quantification of ponatinib fluorescence intensity from simulated single cells was obtained from at least three independent images. For data presentation and statistical analysis, data from all optical fields were pooled. One representative out of at least three images per tissue per animal is depicted.

LD isolation, size determination and high performance liquid chromatography (HPLC)

$1.5x10^7$ NCI-H1703 cells were seeded in T150 cell culture flasks and allowed to adhere. Cells were treated with 10 µM ponatinib for 1 hour. Native LDs were isolated from cells using Cell Biolabs LD isolation kit following the manufacturer's instructions. LD size distribution was determined by dynamic light scattering (DLS) on a Malvern ZetaSizer Nano ZS (Malvern Instruments Ltd., Malvern, UK) equipped with a 4 mW He-Ne, 632.8 nm laser beam at a scattering angle of 173°. Prior to size measurement, LDs were diluted in PBS and measured in triplicates

disposable cuvettes (Brand GmbH + Co KG, Wertheim, Germany). Ponatinib content in LDs was analyzed by HPLC using a Dionex UltiMate 3000 system equipped with an L-7250 injector, an L-7100 pump, an L-7300 column oven (set at 35°C), a D-7000 interface and an L-7400 UV detector (Thermo Fisher Scientific) set at a wavelength of 287 nm. Separation of ponatinib was carried out at 35°C using a Hypersil BDS-C18 column (5 µm, 250 x 4.6 mm I.D., Thermo Fisher Scientific), preceded by a Hypersil BDS-C18 precolumn (5 µm, 10 x 4.6 mm I.D.). The mobile phase consisted of a continuous gradient mixed from ion pair buffer, pH 3.0 (50 mM potassium phosphate with phosphoric acid and 5 mM heptane sulfonic acid) (mobile phase A) and acetonitrile (mobile phase B). Mobile phase was filtered through a 0.45 µM filter (HVLP04700, Millipore, Billerica, MA). Mobile phase B linearly increased from 10% acetonitrile (0 minute) to 95% at 17 minutes, at which point it was kept constant until 20 minutes. The percentage of acetonitrile was decreased within 1 minute to 10% to equilibrate the column for 14 minutes before application of the next sample. Linear calibration curves were performed by spiking drug-free cell culture medium with standard solutions of ponatinib to give a concentration range from 0.1 to 40 µg/mL (average correlation coefficients: >0.999). The limit of quantification (LOQ) for ponatinib was 0.15 µg/ml. Coefficients of accuracy and precision for these compounds were < 9.3%.

Clonogenic assay

$3x10^4$ 3T3-L1/F cells were seeded in 24-well plates, grown to confluency, differentiated (or not) into 3T3-L1/A cells and incubated with indicated concentrations of ponatinib for 24 hours. $2.5x10^3$ NCI-H1703 cells were seeded in 24-well plates and left to adhere overnight. Non-pre-exposed, 3T3-L1/F- or 3T3-L1/A-pre-exposed supernatant was transferred onto cancer cells and incubated for 96 hours. Cells were fixed with methanol and stained with crystal violet. For cell quantification,

crystal violet was re-dissolved overnight in 2% sodium dodecyl sulfate- polyacrylamide (SDS) and pipetted into 96-well plates. Densitometric analysis of crystal violet was performed measuring absorbance at 560 nm on a Tecan Infinite 200 PRO NanoQuant Microplate Reader (Männedorf, Switzerland). Analysis was performed in triplicates and one out of at least three independent experiments is depicted.

Intracellular lipid determination by gas chromatography

$3x10^6$ DMS114 and NCI-H1703 cells were seeded into 10 cm petri dishes and allowed to adhere overnight, followed by incubation in normal media for 72 hours. Ponatinib-selected cells did not receive drug treatment for several weeks prior to the experiment. Parental DMS114 cells were exposed to 0.1 µM ponatinib for 1 hour, followed by a washout and incubation in drug-free media for one week prior to measurement. Cells were detached using trypsin/EDTA, pelleted by centrifugation and stored at -80°C. Cell pellets were re-suspended in 1000 µl aqua dest. and 60 µl were removed for cell protein quantification (lysis in 0.1 M NaOH followed by Bradford-assay). The remaining cell suspension was used for standard FOLCH (chlorophorm : methanol = 2:1) extraction and lipid extracts were subjected to gas chromatography as described [2]. Briefly, triglycerides were quantified using a GC-2010 gas chromatograph (Shimadzu, Kyoto, Japan). Tri-decanoyl glycerol and Tri-nonadecanoyl glycerol (Sigma) were used as standards, respectively. The chromatograms were quantified using GC Solutions 2.3 (Shimadzu), and results were normalized to cell protein.

Intracellular lipidomics analysis

Sample preparation for LC-MS analysis. DMS114 and DMS114/PON cells (2×10^5) seeded in 6-well plates were left untreated, treated with either vehicle (DMSO) or ponatinib (1 µM) for 4 hours in duplicates of duplicates, followed by a change of media. After 72 hours of incubation in drug-free condition, the medium was removed and the cells were gently washed twice with PBS (1×). All PBS was removed and methanol was added including an Equisplash® LIPIDOMIX® quantitative mass spectrometry internal standard in methanolic solution (995 µl MeOH + 5 µl internal standard). The cells were incubated for 30 min at 4 °C in the dark. Thereafter, the cells were scraped and the methanolic solution was transferred into glass vials and stored at –80 °C. Before sample preparation, the glass vials were thawed at 4 °C for 1 hour. Butanol was added (1 ml) and the samples were incubated for 15 min at room temperature and vortexed. An aliquot of each sample (1.5 ml) was transferred to a fresh glass vial, which was centrifuged (2000 rpm, 15 min). The supernatant (1.2 ml) was subsequently transferred into a fresh glass vial and dried under nitrogen at room temperature. Finally, the sample was reconstituted in a mixture of butanol/methanol (1:1 v/v, 50 µl) and analysed by mass spectrometry.

UHPLC-MS. An UHPLC system (Thermo Scientific™ Vanquish, Austria) equipped with a reversed phase C18 column (Kinetex® 2.6 µm XB-C18 100 Å, LC Column 100 x 2.1 mm, Torrance, CA, USA) was used for separation of the analytes. The flow rate was set to 300 µl min^{-1}, the HPLC oven temperature to 50 °C, the auto sampler to 4 °C and the injection volume was 1 µl. A gradient method with a total run time of 25 min was applied using eluent A (H_2O : isopropanol (10:3 v/v) + ammonium formate (10 mM) + 0.1% FA) and eluent B (acetonitrile : isopropanol (1:1 v/v) + ammonium formate (10 mM) + 0.1% FA). The initial LC condition started at 40% B for 1 min. A linear gradient was applied to 75% eluent B within 9 min and to 80% eluent B within 16 min. Then, 90% eluent B was reached after 1 min and finally 100% eluent B within

0.1 min, which was kept at 2.9 min. Finally, the eluent system was restored to the initial conditions within 0.1 min.

A quadrupole-orbitrap mass spectrometer (Thermo Scientific™ QExactive™ HF hybrid quadrupole-orbitrap mass spectrometer) was employed for MS analysis, equipped with a HESI source. Data was collected in the mass range of *m/z* 150–1800 in full scan mode at 120'000 resolution (*m/z* 200) and in positive ionization mode. A Top 5 method was chosen for MS/MS fragmentation in data dependent acquisition (DDA) mode. Fragmentation experiments were performed using HCD with a normalized collision energy of 26 and a resolution of 15'000 (*m/z* 200). Typical instrument parameters were as follows: spray voltage 3.5 kV, capillary temperature 220 °C, sheath gas 30 and auxiliary gas 5 in arbitrary units.

Data Processing. Identification of lipid species was performed by using Lipid Data Analyzer (LDA), Version 2.0[3]. The software uses the retention time, *m/z*-value, intensity and isotopic intensity distributions for peak selection. For the identification, the LDA uses the exact mass of a feature and the corresponding MS/MS fragments applying specific decision rules. In positive ion mode, head groups of lipids and neutral losses are detected and used for assignment of the exact mass to a certain lipid species.

In a second step the software Compound Discoverer 3.1 (Thermo Scientific™, Austria) was used for feature identification (5 ppm), retention time alignment (0.2 min) and identification of lipid species using an in-house built lipid-data base by mzVault (Thermo Scientific™, Austria). Internal normalization was applied using constant sum as the normalization type and data is represented in Log2-space of the normalized area under the curve (nAUC). It was assumed that that similar ionization efficiencies can be expected within a given lipid class and among the different samples. Deuterated internal standards were used as retention time markers. Perseus (Version 2.6.3) was

used to perform the principle component analysis and to generate the heat map. Bar charts were generated using GraphPad Prism (Version 6.07). Lipid names and abbreviations are given according to LIPID MAPS nomenclature and the detected retention times.

Flow cytometry

3-5x10^5 cells were trypsinized and resuspended in serum-free RPMI containing 15 mM 4-(2-hydroxyethyl)piperazine-1-ethanesulfonic acid (HEPES, Sigma) and 2.09 mg/ml 4-morpholine-propanesulfonic acid (MOPS, Sigma). Cells were incubated with the indicated concentrations of ponatinib. Intracellular drug accumulation over time was measured using a LSRFortessa flow cytometer (BD Biosciences, NJ, USA). To analyze intracellular retention kinetics, cells were treated with the indicated ponatinib concentrations for 1 hour and measured at the indicated timepoints following a washout and incubation in drug-free media. Bodipy 493/503 was added to the cells at a final concentration of 1 µM for 15 minutes before measurement. To determine ponatinib uptake from 3T3-L1/F- and 3T3-L1/A-pre-exposed media in cancer cells, confluent 3T3-L1/F and 3T3-L1/A cells were incubated for 24 hours with indicated concentrations of ponatinib (diluted in RPMI containing 10% FCS) in 6-well plates. Subsequently, supernatant was transferred to new 6-well plates containing 5x10^5 adherent NCI-H1703 cells, and incubated for 1 hour, followed by trypsinization and flow cytometric analysis. Ponatinib and Bodipy 493/503 fluorescence was determined using 405/488 nm laser excitation wavelength and Horizon V450 (450/40 nm)/FITC (530/30 nm) bandpass emission filters, respectively. Data were analyzed by FlowJo software (Ashland, OR, USA). Fluorescence intensities are depicted as arbitrary units (a.u.) or as values relative to respective untreated controls.

Western blot analysis

5x10^5 cells were seeded in 6-well plates and allowed to adhere. For FGFR1 signaling experiments, cells were treated with 1 µM for 1 hour or with 20 ng/ml bFGF for 5 min, followed by media removal and cell harvesting in ice-cold PBS. Alternatively, cells were pretreated with 100 µM OA or 0.5 µM TC for 72 hours, followed by exposure to the indicated concentrations of ponatinib. For experiments using 3T3-L1/F- and 3T3-L1/A-pre-exposed media in cancer cells, confluent 3T3-L1/F and 3T3-L1/A cells were incubated for 24 hours with indicated concentrations of ponatinib (diluted in RPMI containing 10% FCS) in 6-well plates. Subsequently, supernatant was transferred to new 6-well plates containing 5x10^5 adherent NCI-H1703 or DMS114 cells, and incubated for 1 hour, followed by trypsinization and whole cell protein isolation. Sodium dodecyl sulfate-polyacrylamide gel electrophoresis (SDS-PAGE) was performed to separate whole-cell protein extracts. Proteins were transferred onto polyvinylidene difluoride membranes (PVDF, Thermo Fisher Scientific, Waltham, MA, USA). Anti-FGFR1 (D8E4), Anti-AKT (11E7), anti-phospho-AKT (D9E), ERK1/2, phospho-ERK1/2 (Thr202/Tyr204), anti-CHOP (L63F7), and anti-BiP (C50B12) antibodies were purchased from Cell Signaling Technology (Danvers, MA, USA). Anti-ß-actin (AC-15) was obtained from Sigma. Anti-ADRP (B-6), anti-Tip47 (F-10) antibodies as well as horseradish peroxidase (HRP)-coupled secondary antibodies were purchased from Santa Cruz Biotech (Dallas, TX, USA).

Live cell microscopy

3x10^4 NCI-H1703 cells were seeded in 8-well culture slides (Ibidi, Martinsried, Germany) and allowed to adhere overnight. Subsequently, cells were incubated with the indicated concentrations of ponatinib. Drug accumulation was imaged at the indicated timepoints on a Visitron Systems

live cell microscope (Puchheim, Germany) using a 40x immersion oil lens. To assess ponatinib retention kinetics, ponatinib was removed from the cells after 1 hour, followed by incubation in drug-free media and imaging at the indicated timepoints. Ponatinib was imaged by 395/25 nm LED excitation and a 460/50 nm bandpass emission filter (Visitron Systems). Data were analyzed using VisiView (Visitron Systems) and ImageJ softwares.

Confocal fluorescence microscopy

$5x10^3$ cells were plated in 8-well chamber slides (Ibidi) and allowed to adhere overnight. Cells were coincubated for 1 hour with 10 µM of ponatinib and 1 µM Lysotracker® Red. Bodipy 493/503 staining of ponatinib-treated cells was performed 10 minutes prior to fixation at a final concentration of 1 µM. Cells were fixed with 4% paraformaldehyde (PFA) for 15 min. Images were acquired using an inverted point scanning confocal microscope (LSM700, Zeiss, Jena, Germany) with a 63x oil immersion objective and Zen2010 software (Zeiss). Ponatinib, LysoTracker® Red/mCherry and Bodipy 493/503 were detected using 405 nm, 555 nm and 488 nm solid state laser lines and 420 nm longpass, 556 nm shortpass and 559 nm longpass emission filters, respectively. Thresholded MCC was calculated using ImageJ software to determine colocalization of ponatinib- and LysoTracker® Red or Bodipy 493/503-derived signals. A MCC value of 1 describes perfect colocalization, 0 means no colocalization [4]. To obtain a mean thresholded MCC, ten to twenty individual cells were analyzed from a minimum of three independent micrographs. Costes Colocalizaiton Test [5] was applied using ImageJ software to determine the statistical significance of overlapping pixel intensities. A p-value of 0.95 or greater was considered statistically significant. For imaging of fibroblast/adipocyte - cancer cell co-cultures, $3x10^4$ 3T3-L1/F cells were seeded into 8-well chamber slides (Ibidi), left to grow to full

adherence, and differentiated (or not) into 3T3-L1/A cells. $1.5x10^4$ NCI-H1703/mCherry cells were seeded on top of 3T3-L1/F or 3T3-L1/A cells and left to adhere overnight. Cells were incubated with indicated ponatinib concentrations for 1 hour, and with 0.5 µM Bodipy 493/503 prior to fixation. Confocal images were obtained using a 63x immersion oil objective. Ponatinib fluorescence intensity in cancer cells was quantified using ImageJ. Cancer cells were delineated by creating regions of interest (ROI) around mCherry-positive areas. Ponatinib fluorescence was determined by measuring blue pixel intensity for each ROI in drug-treated 3T3-L1F- or 3T3-L1/A-cancer cell co-cultures and by subtracting background fluorescence in ROIs of respective untreated control images. Graphs were plotted using GraphPad Prism software, pooling all mean ROI intensities of at least three independent images.

Quantitative real-time PCR (qPCR)

DMS114 and NCI-H1703 cells were seeded in T25 flasks and either left untreated or pretreated with 100 µM OA for 72 hours. Cells were seeded in 6 well plates ($2.5x10^5$), left to adhere, and treated with 0.25 µM (NCI-H1703) or 1 µM (DMS114) ponatinib for 1 hour, followed by washout and incubation for 72 hours in drug-free media. Total RNA was isolated from cell lysates using Trizol reagent (Life Technologies, Carlsbad, CA, USA). cDNA was generated using MMLV reverse transcriptase (Thermo Fisher Scientific). PCR was perfomed using the GoTaq protocol (Promega, Madison, WI, USA) and the following primers: *PLIN1* sense: 5′-CCTGCCTTACATGGCTTGTT-3′, *PLIN1* antisense: 5′-CCTTTGTTGACTGCCATCCT-3′, *PLIN2* sense 5′-GGCTAGACAGGATTGAGGAGAG-3′, *PLIN2* antisense 5′-TCACTGCCCCTTTGGTCTTG-3′, *PLIN3* sense 5′-GGTCCTAAGCCTGATGGAAA-3′, *PLIN3* antisense 5′-CTGGCCTTCCACCAGCTTCT-3′, *ACTB* sense: 5′-

GGATGCAGAAGGAGATCACTG-3′, *ACTB* antisense: 5′-CGATCCACACGGAGTACTTG-3′. *ACTB* served as internal control. *PLIN1, PLIN2, PLIN3* expression levels were normalized to *ACTB* cycle thresholds (ΔCt) of respective cell lines and depicted relative to corresponding untreated controls (1/ΔCT (log2)).

Table S1. The significantly enriched REACTOME gene ontology term 'CHYLOMICRON MEDIATED LIPID TRANSPORT' in ponatinib-selected DMS114 cells. Significant enrichment of lipid homeostasis-associated genes differentially expressed in ponatinib-selected as compared to treatment-naïve DMS114 cells was determined by GSEA of genome-wide mRNA expression analysis with a false discovery rate (FDR) cut-off <0.25.

REACTOME_CHYLOMICRON_MEDIATED_LIPID_TRANSPORT					
GENE SYMBOL	GENE_TITLE	RANK IN GENE LIST	RANK METRIC SCORE	RUNNING ES	CORE ENRICHMENT
LDLRAP1	low density lipoprotein receptor adaptor protein 1	4192	0.060583174	-0.12893835	No
P4HB	procollagen-proline, 2-oxoglutarate 4-dioxygenase (proline 4-hydroxylase), beta polypeptide	4885	0.051232234	-0.12782194	No
HSPG2	heparan sulfate proteoglycan 2 (perlecan)	4924	0.050851811	-0.101661235	No
LDLR	low density lipoprotein receptor (familial hypercholesterolemia)	6905	0.03258476	-0.16040576	No
APOC2	apolipoprotein C-II	8139	0.024236036	-0.19484445	No
APOA2	apolipoprotein A-II	9723	0.0153013	-0.24765094	No
LIPC	lipase, hepatic	9961	0.013958351	-0.24921791	No
APOA5	apolipoprotein A-V	11103	0.008404001	-0.28870606	No
APOB	apolipoprotein B (including Ag(x) antigen)	13146	-7.70E-04	-0.3671292	No
APOC3	apolipoprotein C-III	17348	-0.020179864	-0.5183662	No
SAR1B	SAR1 gene homolog B (S. cerevisiae)	17401	-0.020446954	-0.50926507	No
APOA1	apolipoprotein A-I	17581	-0.021419449	-0.5045391	No
LPL	lipoprotein lipase	20058	-0.038923446	-0.57899034	No
SDC1	syndecan 1	24523	-0.147017255	-0.6714708	Yes
APOE	apolipoprotein E	25644	-0.337299705	-0.53145856	Yes
MTTP	microsomal triglyceride transfer protein	25898	-0.997389138	6.56E-04	Yes

Table S2. The significantly enriched REACTOME gene ontology term 'LIPID DIGESTION, MOBILIZATION, AND TRANSPORT' in ponatinib-selected DMS114 cells. Significant enrichment of lipid homeostasis-associated genes differentially expressed in ponatinib-selected as compared to treatment-naïve DMS114 cells was determined by GSEA of genome-wide mRNA expression analysis with a false discovery rate (FDR) cut-off <0.25.

REACTOME_LIPID_DIGESTION_MOBILIZATION_AND_TRANSPORT					
GENE SYMBOL	GENE_TITLE	RANK IN GENE LIST	RANK METRIC SCORE	RUNNING ES	CORE ENRICHMENT
ALB	albumin	414	0.278376639	0.057067614	No
PRKACB	protein kinase, cAMP-dependent, catalytic, beta	1477	0.14041838	0.05287571	No
PPP1CB	protein phosphatase 1, catalytic subunit, beta isoform	1818	0.122751296	0.07195412	No
PNLIPRP2	pancreatic lipase-related protein 2	2863	0.087050959	0.054449756	No
A2M	alpha-2-macroglobulin	3345	0.076028571	0.05581398	No
LDLRAP1	low density lipoprotein receptor adaptor protein 1	4192	0.060583174	0.03901553	No
P4HB	procollagen-proline, 2-oxoglutarate 4-dioxygenase (proline 4-hydroxylase), beta polypeptide	4885	0.051232234	0.025715202	No
HSPG2	heparan sulfate proteoglycan 2 (perlecan)	4924	0.050851811	0.037594285	No
ABCG5	ATP-binding cassette, sub-family G (WHITE), member 5 (sterolin 1)	5512	0.044495653	0.026584292	No
ABCG8	ATP-binding cassette, sub-family G (WHITE), member 8 (sterolin 2)	5813	0.041629273	0.025915416	No

SCARB1	scavenger receptor class B, member 1	6533	0.035309803	0.007392024	No
LDLR	low density lipoprotein receptor (familial hypercholesterolemia)	6905	0.03258476	0.0016047	No
CETP	cholesteryl ester transfer protein, plasma	7439	0.028604889	-0.011489124	No
FABP4	fatty acid binding protein 4, adipocyte	7658	0.027185785	-0.012779648	No
APOC2	apolipoprotein C-II	8139	0.024236036	-0.02497161	No
APOA2	apolipoprotein A-II	9723	0.0153013	-0.082143426	No
LIPC	lipase, hepatic	9961	0.013958351	-0.08764039	No
PRKACG	protein kinase, cAMP-dependent, catalytic, gamma	10118	0.01316408	-0.09021491	No
BMP1	bone morphogenetic protein 1	10661	0.0105207	-0.10840347	No
APOA5	apolipoprotein A-V	11103	0.008404001	-0.123243645	No
CUBN	cubilin (intrinsic factor-cobalamin receptor)	11480	0.00656822	-0.13605322	No
ABHD5	abhydrolase domain containing 5	12740	9.60E-04	-0.18446565	No
CLPS	colipase, pancreatic	12832	5.19E-04	-0.1878469	No
PPP1CC	protein phosphatase 1, catalytic subunit, gamma isoform	12871	3.99E-04	-0.18921086	No
APOB	apolipoprotein B (including Ag(x) antigen)	13146	-7.70E-04	-0.19959965	No
PRKACA	protein kinase, cAMP-dependent, catalytic, alpha	15030	-0.009234112	-0.26996002	No
LIPE	lipase, hormone-sensitive	16732	-0.016882051	-0.331278	No
APOC3	apolipoprotein C-III	17348	-0.020179864	-0.34975284	No
SAR1B	SAR1 gene homolog B (S. cerevisiae)	17401	-0.020446954	-0.34639576	No
AMN	amnionless homolog (mouse)	17535	-0.021187376	-0.34597528	No
APOA1	apolipoprotein A-I	17581	-0.021419449	-0.34209237	No
null	null	18300	-0.025711956	-0.3630964	No
PNLIP	pancreatic lipase	19916	-0.037629262	-0.41564432	No
PPP1CA	protein phosphatase 1, catalytic subunit, alpha isoform	19958	-0.03786372	-0.4072904	No
LPL	lipoprotein lipase	20058	-0.038923446	-0.40090021	No
MGLL	monoglyceride lipase	20729	-0.045366723	-0.41488978	No
LPA	lipoprotein, Lp(a)	21610	-0.055776391	-0.43426415	No
ABCG1	ATP-binding cassette, sub-family G (WHITE), member 1	23316	-0.093890987	-0.47552297	Yes
ABCA1	ATP-binding cassette, sub-family A (ABC1), member 1	23900	-0.114886045	-0.46790183	Yes
SDC1	syndecan 1	24523	-0.147017255	-0.45335415	Yes
PLTP	phospholipid transfer protein	24529	-0.147335827	-0.41487375	Yes
LCAT	lecithin-cholesterol acyltransferase	24711	-0.160610929	-0.3797118	Yes
CAV1	caveolin 1, caveolae protein, 22kDa	25522	-0.288764358	-0.33522427	Yes
APOE	apolipoprotein E	25644	-0.337299705	-0.25136474	Yes
MTTP	microsomal triglyceride transfer protein	25898	-0.997389138	6.57E-04	Yes

Table S3. Relative concentrations of major cholesterol and triacylglyceride species in untreated, ponatinib-pretreated, and ponatinib-selected DMS114 as well as NCI-H1703 cells. Concentrations of individual lipid species were determined by gas chromatography and are given as mean ± SD of triplicate values (µg lipid / mg total cell protein). For detailed experimental conditions, see Materials and Methods section.

**Lipid species (µg/mg protein)**	**unesterified cholesterol**	**CE16**	**CE18**	**CE20**	**total cholesterol**	**TG50**	**TG52**	**TG54**	**total triglycerides**
**DMS114**									
**untreated**	20.742 ± 0.887	0.880 ± 0.226	1.883 ± 0.223	1.049 ± 0.078	24.555 ± 1.414	2.278 ± 0.379	2.960 ± 0.129	0.662 ± 0.087	6.265 ± 0.689
**Ponatinib-pretreated**	33.538 ± 7.227	1.787 ± 0.275	3.161 ± 0.499	0.599 ± 0.197	39.084 ± 8.070	5.271 ± 1.819	6.557 ± 1.486	3.154 ± 0.538	14.981± 3.830
**Ponatinib-selected**	33.265 ± 2.304	1.130 ± 0.034	2.273 ± 0.110	1.092 ± 0.161	37.760 ± 2.475	7.531 ± 0.525	8.674 ± 0.912	4.364 ± 0.196	20.836 ± 1.623
**NCI-H1703**									
**untreated**	15.728 ± 1.962	3.249 ± 0.408	17.913 ± 2.103	3.010 ± 0.363	39.900 ± 4.713	3.520 ± 0.497	7.207 ± 0.815	2.639 ± 0.037	14.237 ± 1.336
**Ponatinib-pretreated**	14.084 ± 1.651	1.650 ± 0.200	15.448 ± 1.871	2.411 ± 0.228	33.593 ± 3.871	2.924 ± 0.209	7.175 ± 0.577	5.100 ± 0.175	16.115 ± 0.687
**Ponatinib-selected**	16.978 ± 4.545	1.698 ± 0.484	10.417 ± 2.528	1.129 ± 0.273	30.22151 ± 7.823	4.296 ± 0.858	10.304 ± 2.404	8.019 ± 2.311	23.713 ± 5.672

CE, cholesterol ester; TG, triacylglyceride;

Figure S1

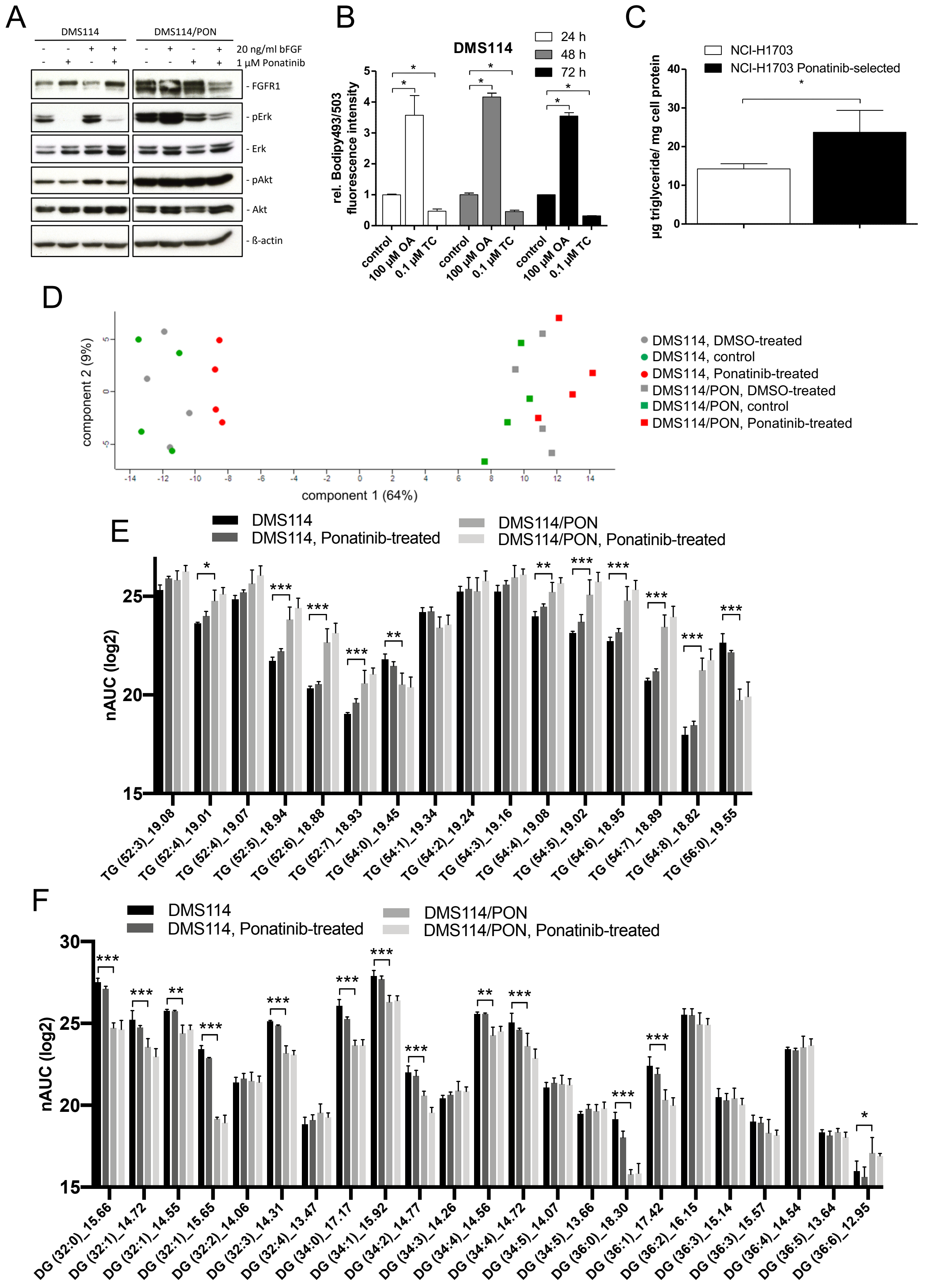
A
DMS114
DMS114/PON
20 ng/ml bFGF
1 µM Ponatinib
- FGFR1
- pErk
- Erk
- pAkt
- Akt
- ß-actin
B
DMS114
24 h
48 h
72 h
rel. Bodipy493/503 fluorescence intensity
control
100 µM OA
0.1 µM TC
C
NCI-H1703
NCI-H1703 Ponatinib-selected
µg triglyceride/ mg cell protein
D
component 2 (9%)
component 1 (64%)
DMS114, DMSO-treated
DMS114, control
DMS114, Ponatinib-treated
DMS114/PON, DMSO-treated
DMS114/PON, control
DMS114/PON, Ponatinib-treated
E
DMS114
DMS114, Ponatinib-treated
DMS114/PON
DMS114/PON, Ponatinib-treated
nAUC (log2)
TG (52:3)_19.08
TG (52:4)_19.01
TG (52:4)_19.07
TG (52:5)_18.94
TG (52:6)_18.88
TG (52:7)_18.93
TG (54:0)_19.45
TG (54:1)_19.34
TG (54:2)_19.24
TG (54:3)_19.16
TG (54:4)_19.08
TG (54:5)_19.02
TG (54:6)_18.95
TG (54:7)_18.89
TG (54:8)_18.82
TG (56:0)_19.55
F
DMS114
DMS114, Ponatinib-treated
DMS114/PON
DMS114/PON, Ponatinib-treated
nAUC (log2)
DG (32:0)_15.66
DG (32:1)_14.72
DG (32:1)_14.55
DG (32:1)_15.65
DG (32:2)_14.06
DG (32:3)_14.31
DG (32:4)_13.47
DG (34:0)_17.17
DG (34:1)_15.92
DG (34:2)_14.77
DG (34:3)_14.26
DG (34:4)_14.56
DG (34:4)_14.72
DG (34:5)_14.07
DG (34:5)_13.66
DG (36:0)_18.30
DG (36:1)_17.42
DG (36:2)_16.15
DG (36:3)_15.14
DG (36:3)_15.57
DG (36:4)_14.54
DG (36:5)_13.64
DG (36:6)_12.95

Figure S1. Selection for ponatinib resistance results in elevated triglyceride levels in FGFR1-driven lung cancer cells. A. Phosphorylation levels of FGFR downstream effectors in parental and drug-selected DMS114 cells upon exposure to 20 ng/ml bFGF for 5 min or to 1 µM ponatinib for 1 hour. ß-actin served as loading control. **B**. Impact of 72 hours treatment with 100 µM OA/0.5 µM TC on intracellular lipid content was determined by flow cytometry at the indicated timepoints after staining with 1 µM Bodipy 493/503. Values are shown relative to the untreated control. * $p<0.05$, two-tailed student's t-test. **C**. Triglyceride concentrations in ponatinib-selected versus unselected NCI-H1703 cells were determined by gas chromatography. One representative experiment, performed in triplicates, is shown. * $p<0.05$, two-tailed student's t-test. $t=2.816$, $DF=4$; **D.** Principal component analysis of the intensity distribution of 135 identified lipids from whole-cell extracts of DMS114 (circles) and DMS114/PON (squares) cells either untreated (green), vehicle-treated (DMSO, grey) or treated with ponatinib (red). **E.** Bar charts of 16 identified triglycerides (TGs) and their normalized intensities, including their total number of carbon atoms in the three fatty acid chains and number of double bonds in parentheses, as well as their retention times in minutes. The standard deviations represent four biological replicates (duplicates of duplicates). Statistical significance levels of differences between lipid levels of untreated DMS114 as compared to DMS114/PON cells are depicted. * $p<0.05$, ** $p<0.01$, *** $p<0.001$, two-way ANOVA, Bonferroni post-test. **F.** Bar charts of the 23 identified diglycerides (DGs) and their normalized intensities, including their total number of carbon atoms in the two fatty acid chains and number of double bonds in parentheses, as well as their retention times in minutes. The standard deviations represent four biological replicates (duplicates of duplicates). Statistical significance levels of differences between lipid levels of untreated DMS114 as compared to

DMS114/PON cells are depicted. * $p<0.05$, ** $p<0.01$, *** $p<0.001$, two-way ANOVA, Bonferroni post-test.

Figure S2

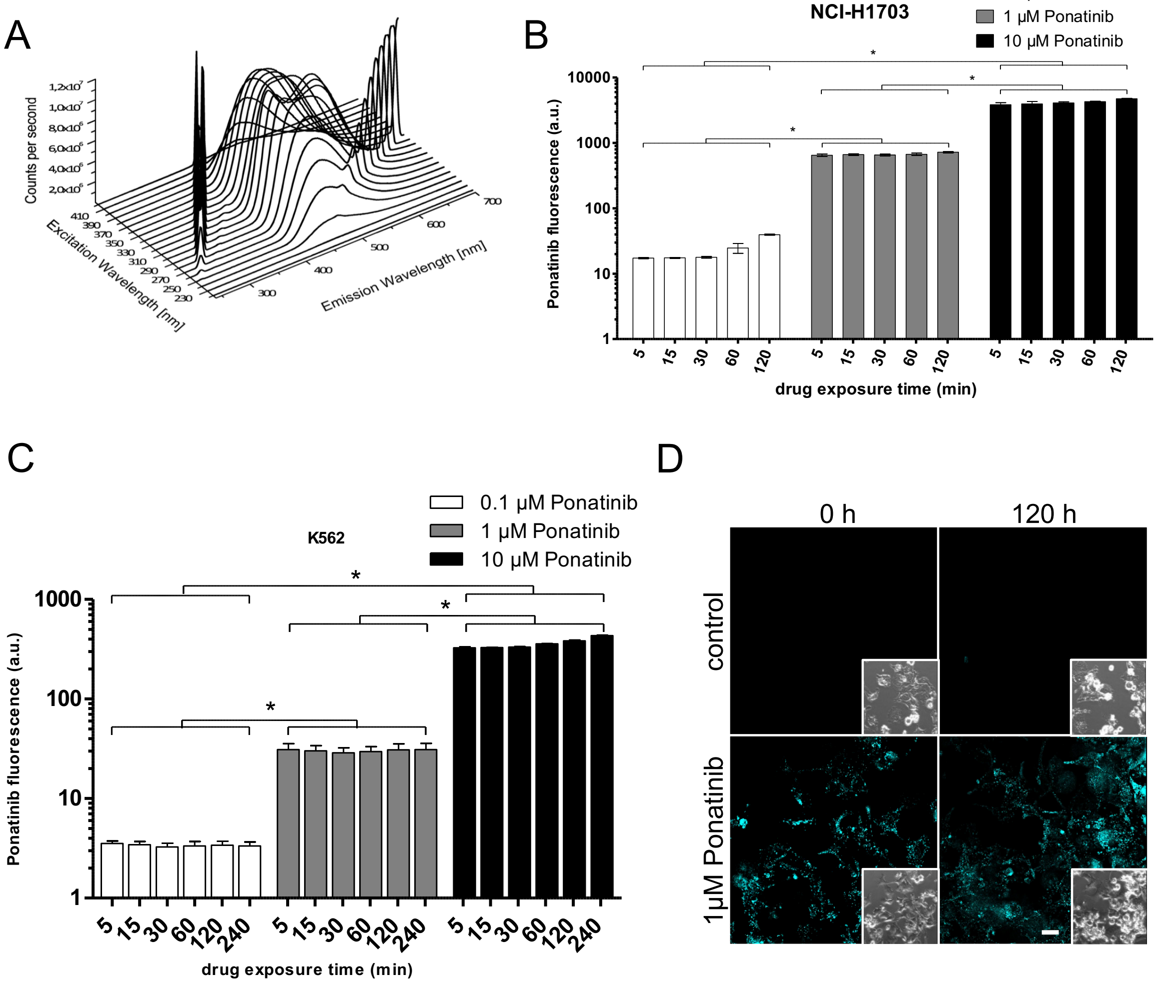

Figure S2. Intrinsic ponatinib fluorescence properties enable monitoring of drug uptake and retention kinetics. A. Cell-free fluorescence activity of ponatinib was determined by fluorescence spectroscopy, yielding a 3-dimensional, full excitation-emission landscape. Spectra are depicted for an excitation wavelength range between 220 nm and 420 nm. Emission was recorded from 240 nm to 700 nm. Rayleigh scattering of 1st and 2nd order appear as diagonal ridges. **B**, **C**. Uptake kinetics in NCI-H1703 (b) and K562 (c) cells treated with increasing ponatinib concentrations was measured by flow cytometry at the indicated time points. Fluorescence signals are depicted as arbitrary units (a.u.). The asterisks indicate statistical significance of all time-points between each drug concentration. One representative experiment, performed in biological triplicates, is shown

out of three replicates. * $p<0.05$, two-way ANOVA, Bonferroni post-test. (B) $F=3031$, DFgroup=2, DFresidual=15; (C) $F=2297$, $DF_{group}=2$, $DF_{residual}=18$; **D**. Ponatinib retention in NCI-H1703 cells was analyzed by live cell microscopy. Cells were treated for 1 hour with 1 μM of ponatinib, followed by incubation in drug-free media. Ponatinib is pseudo-colored in cyan. The scale bar indicates 10 μm.

Figure S3

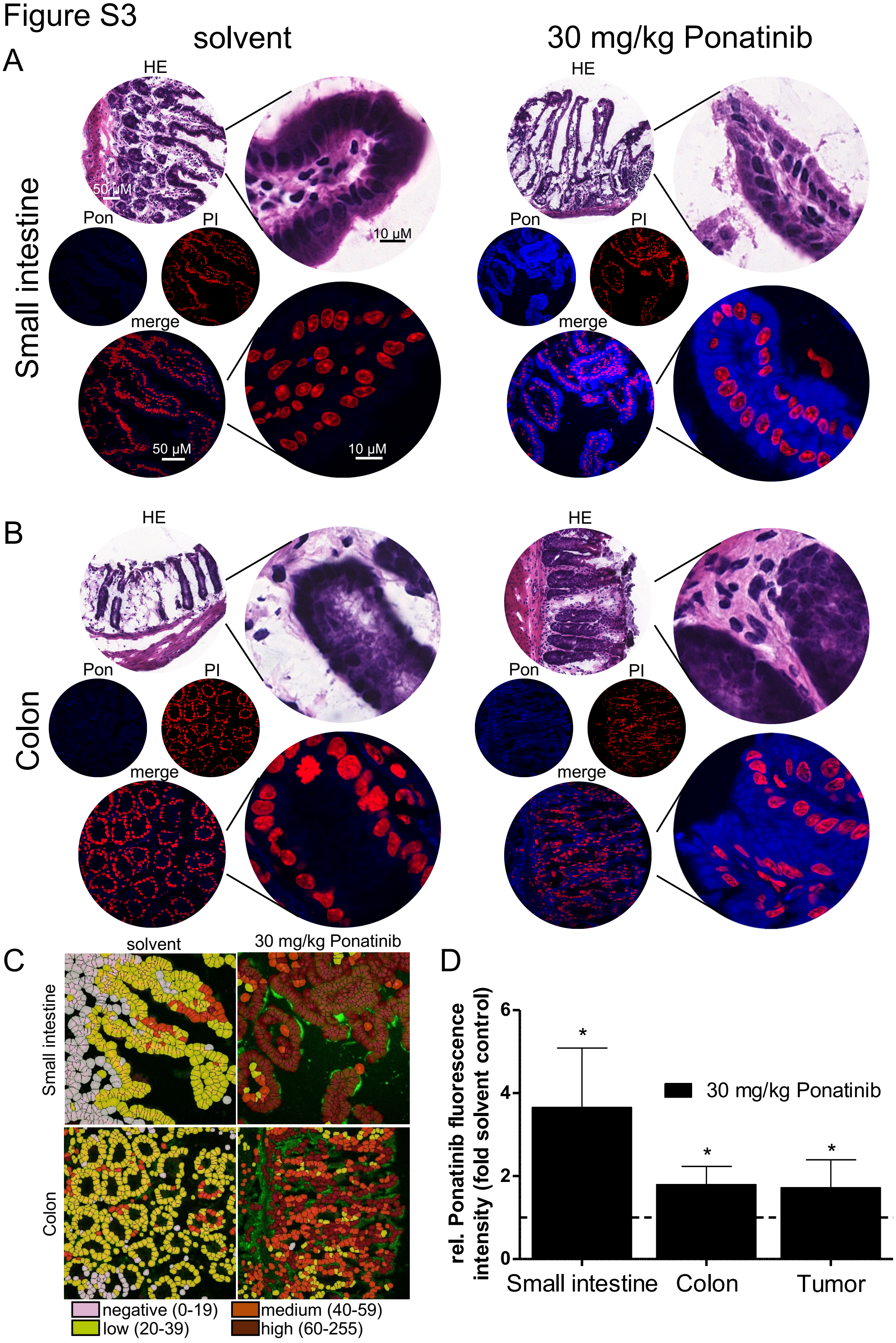
solvent
30 mg/kg Ponatinib
A
Small intestine
HE
50 µM
10 µM
Pon
PI
merge
50 µM
10 µM
B
Colon
HE
Pon
PI
merge
C
solvent
30 mg/kg Ponatinib
Small intestine
Colon
negative (0-19)
low (20-39)
medium (40-59)
high (60-255)
D
rel. Ponatinib fluorescence intensity (fold solvent control)
0
2
4
6
30 mg/kg Ponatinib
Small intestine
Colon
Tumor
*

Figure S3. Ponatinib imaging in tissue cryosections from orally-treated mice. Ponatinib fluorescence imaging in cryosections of (**A**) small intestine and (**B**) colon by confocal fluorescence microscopy. Mice bearing subcutaneous A549 tumor xenografts received a single oral dose of 30 mg ponatinib per kg bodyweight or solvent. 2 h after drug administration, mice were sacrificed and consecutive cryosections of OCT-embedded tissues were generated. PI served as nuclear counterstain. Representative micrographs of specimen are shown from the experiment, performed in duplicates. Histology of consecutive tissue sections was visualized by hematoxylin and eosin staining. One representative out of at least three images per tissue per animal is depicted. **C**. Simulation of single cells in tissue sections was performed using Tissue Studio software. For illustration of single-cell drug fluorescence intensity levels, the ponatinib channel of 8-bit images was divided and colored according to increasing intensity ranges (values are indicated below the images). **D**. Quantification of drug fluorescence intensity from simulated single cells of small intestine- ($n_{solvent}$=1281, $n_{treated}$=877), colon-, as well as tumor sections was obtained from at least three independent images in each tissue slice of all mice. For data presentation and statistical analysis, data from all optical fields were pooled. Values are shown normalized to respective solvent controls. * $p<0.05$, D'Agostino and Pearson omnibus normality test, followed by two-tailed Mann-Whitney test.

Figure S4

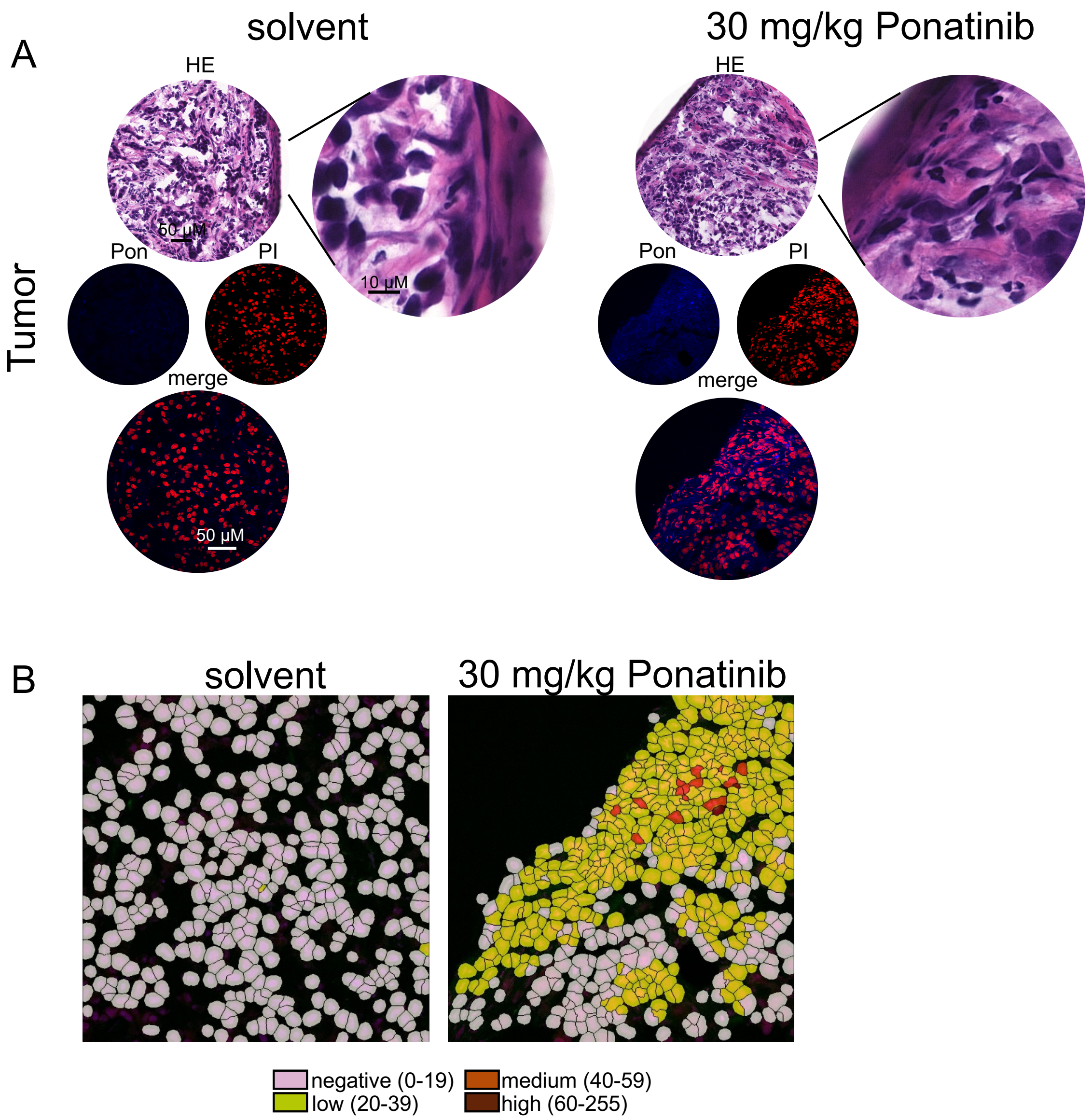

Figure S4. Ponatinib imaging in tumor cryosections from orally-treated mice. A. Ponatinib fluorescence imaging in tumor cryosections by confocal fluorescence microscopy. Mice bearing subcutaneous A549 tumor xenografts received a single oral dose of 30 mg ponatinib per kg bodyweight or solvent. 2 h after drug administration, mice were sacrificed and consecutive cryosections of OCT-embedded tumors were generated. PI served as nuclear counterstain.

Representative micrographs of specimen are shown from the experiment, performed in duplicates. Histology of consecutive tissue sections was visualized by hematoxylin and eosin staining. **B**. Simulation of single cells in tumor sections was performed using Tissue Studio software. For illustration of single-cell drug fluorescence intensity levels, the ponatinib channel of 8-bit images as divided and colored according to increasing intensity ranges (values are indicated below the images).

Figure S5

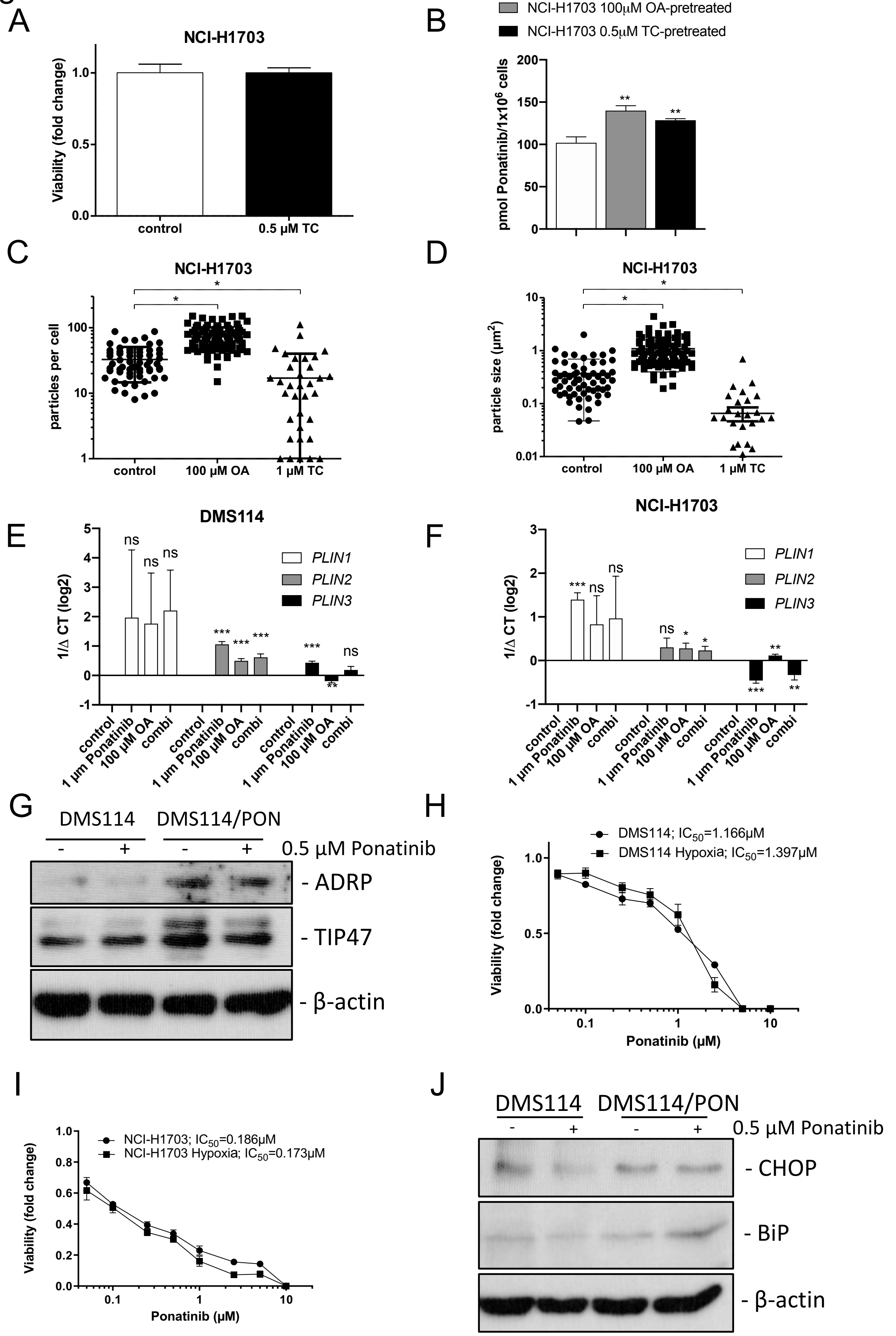
A
NCI-H1703
Viability (fold change)
control
0.5 µM TC
B
NCI-H1703
NCI-H1703 100µM OA-pretreated
NCI-H1703 0.5µM TC-pretreated
pmol Ponatinib/1x10^6 cells
C
NCI-H1703
particles per cell
control
100 µM OA
1 µM TC
D
NCI-H1703
particle size (µm^2)
control
100 µM OA
1 µM TC
E
DMS114
1/Δ CT (log2)
PLIN1
PLIN2
PLIN3
control
1 µm Ponatinib
100 µM OA
combi
F
NCI-H1703
1/Δ CT (log2)
PLIN1
PLIN2
PLIN3
G
DMS114
DMS114/PON
0.5 µM Ponatinib
- ADRP
- TIP47
- β-actin
H
DMS114; IC50=1.166µM
DMS114 Hypoxia; IC50=1.397µM
Viability (fold change)
Ponatinib (µM)
I
NCI-H1703; IC50=0.186µM
NCI-H1703 Hypoxia; IC50=0.173µM
Viability (fold change)
Ponatinib (µM)
J
DMS114
DMS114/PON
0.5 µM Ponatinib
- CHOP
- BiP
- β-actin

Figure S5. Cancer cell treatment with OA/TC impacts on cancer cellular LD load. A. Impact of 72 hours incubation with 0.5 µM TC on cell viability of NCI-H1703 cells was analyzed by MTT assay. **B.** Total intracellular ponatinib concentrations were quantified by HPLC of NCI-H1703 cells that were 1) untreated, 2) 72 hours pretreated with 100 µM OA, or 3) 72 hours pretreated with 0.5 µM TC. Before cell harvesting and lysis, cells were treated with 10 µM ponatinib for 1 hour. ** $p<0.01$, two-tailed student's t-test. **C, D.** Determination of LD number (C) and size (D) of confocal micrographs of Bodipy 493/503-stained NCI-H1703 cells using ImageJ software-based particle analysis. For each experimental condition, individual cells from at least three independent micrographs were analyzed. * $p<0.05$, D'Agostino and Pearson omnibus normality test, followed by two-tailed Mann-Whitney test. $n_{control}=61$, $n_{OA}=99$, $n_{TC}=39$; **E**, **F**. mRNA expression levels of *PLIN1*, *PLIN2*, and *PLIN3* in DMS114 (E) and NCI-H1703 cells (F) that were either untreated or pretreated with 100 µM OA for 72 h, followed by treatment with 1 µM (DMS114) or 0.25 µM (NCI-H1703) ponatinib for 1 hour and 72 h incubation in drug-free medium, analyzed by qPCR. Values are given normalized to respective untreated controls. * $p<0.05$, ** $p<0.01$, *** $p<0.001$. two-tailed student's t-test. **G.** Expression levels of ADRP (*PLIN2*) and Tip47 (*PLIN3*) in DMS114 and DMS114/PON cells, treated with 0.5 µM ponatinib for 1 hour, analyzed by Western blot. ß-actin served as loading control. **H, I.** Viability of DMS114 (H) and NCI-H1703 (I) cells under normoxic and hypoxic conditions upon 72 h treatment with increasing ponatinib concentrations was determined by MTT assay. **J.** Expression of ER stress markers CHOP and BiP in DMS114 and DMS114/PON cells, treated with 0.5 µM ponatinib for 1 h, analyzed by Western blot. ß-actin served as loading control.

Figure S6

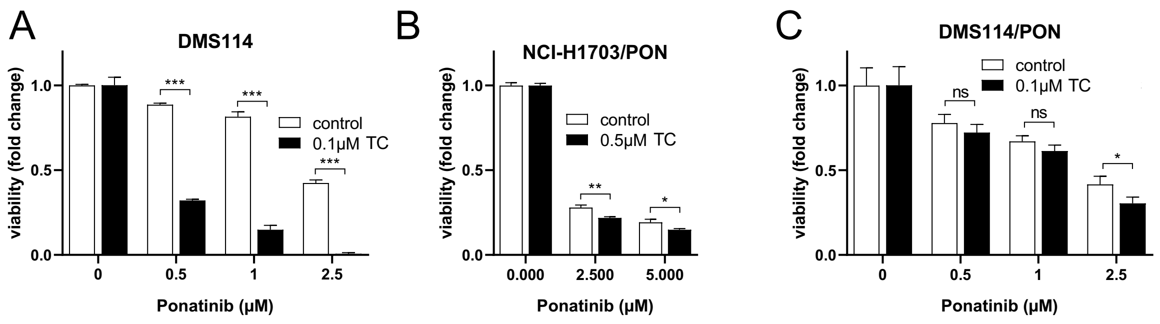

Figure S6. LD depletion sensitizes drug-selected cancer cells towards ponatinib. A-C. Impact of 72 hours (A) or 6 hours (B, C) preincubation with indicated concentrations of TC on cell viability of DMS114 (A), NCI-H1703/PON (B), and DMS114/PON cells (C), treated for 72 hours with indicated concentrations of ponatinib was analyzed by MTT assay. Asterisks indicate levels of significance of difference at respective ponatinib concentrations between TC-pretreated and non-pretreated cells. * $p<0.05$, ** $p<0.01$, *** $p<0.001$, two-ways student's t-test; ns, non-significant;

References

1. Workman P, Aboagye EO, Balkwill F, Balmain A, Bruder G, Chaplin DJ, Double JA, Everitt J, Farningham DA, Glennie MJ, Kelland LR, Robinson V, Stratford IJ, Tozer GM, Watson S, Wedge SR, Eccles SA, Committee of the National Cancer Research I. Guidelines for the welfare and use of animals in cancer research. *Br J Cancer* 2010;**102**: 1555-77.

2. Lohninger A, Preis P, Linhart L, Sommoggy SV, Landau M, Kaiser E. Determination of plasma free fatty acids, free cholesterol, cholesteryl esters, and triacylglycerols directly from total lipid extract by capillary gas chromatography. *Anal Biochem* 1990;**186**: 243-50.

3. Hartler J, Triebl A, Ziegl A, Trotzmuller M, Rechberger GN, Zeleznik OA, Zierler KA, Torta F, Cazenave-Gassiot A, Wenk MR, Fauland A, Wheelock CE, Armando AM, Quehenberger O, Zhang Q, Wakelam MJO, Haemmerle G, Spener F, Kofeler HC, Thallinger GG. Deciphering lipid structures based on platform-independent decision rules. *Nat Methods* 2017;**14**: 1171-4.

4. Dunn KW, Kamocka MM, McDonald JH. A practical guide to evaluating colocalization in biological microscopy. *Am J Physiol Cell Physiol* 2011;**300**: C723-42.

5. Costes SV, Daelemans D, Cho EH, Dobbin Z, Pavlakis G, Lockett S. Automatic and quantitative measurement of protein-protein colocalization in live cells. *Biophys J* 2004;**86**: 3993-4003.
